# Flap Monitoring Techniques: A Review

**DOI:** 10.3390/jcm13185467

**Published:** 2024-09-14

**Authors:** Ignacy Rogoń, Agnieszka Rogoń, Mariusz Kaczmarek, Adam Bujnowski, Jerzy Wtorek, Filip Lachowski, Jerzy Jankau

**Affiliations:** 1 Biomedical Engineering Department, Faculty of Electronics, Telecommunication and Informatics, Gdansk University of Technology, 80-233 Gdansk, Poland; markaczm@eti.pg.edu.pl (M.K.); adabujno@pg.edu.pl (A.B.); jerwtore@pg.edu.pl (J.W.); 2 St. Adalbert’s Hospital, 80-462 Gdansk, Poland; arogon@copernicus.gda.pl; 3 BioTechMed Center, Gdansk University of Technology, 80-233 Gdansk, Poland; 4 Plastic Surgery Division, Medical Univeristy of Gdansk, 80-210 Gdansk, Poland; filiplachowski@gumed.edu.pl (F.L.); jjankau@gumed.edu.pl (J.J.)

**Keywords:** tissue vitality, flap monitoring, postoperative care, perfusion, blood flow

## Abstract

Postoperative tissue flap vitality monitoring enables early detection of clinical complications, allowing for intervention. Timely re-operation can prevent the need for extensive correction procedures, thus reducing healthcare costs and hospitalization time. Statistics show that monitoring can increase the success rate of flap survival to 95% or higher. However, despite the significant progress in monitoring techniques, major and minor complications, leading to the loss of the flap, still occur. This clinical application review aims to provide a comprehensive overview of the recent advancements and findings in flap surgery reconstructions, transplants, and systems for their postoperative assessment. The literature from the years 1925 to 2024 has been reviewed to capture previous and current solutions for monitoring flap vitality. Clinically acclaimed methods and experimental techniques were classified and reviewed from a technical and clinical standpoint. Physical examination, metabolism change, ultrasound method, and electromagnetic (EM) radiation-based measurement methods were carefully evaluated from the perspective of their considered applications. Guidelines aiding engineers in the future design and development process of monitoring systems were proposed. This paper provides a comprehensive overview of the monitoring techniques used in postoperative flap vitality monitoring. It also gives an overview of each approach and potential ways for future development.

## 1. Introduction

Widely used in plastic and reconstructive surgery, flap surgery is a technique where any tissue is removed from a donor site and transferred to a recipient site with an intact blood supply. This procedure is utilized to repair more complicated anatomic features like the jaw or breast or to fill a deficiency like a wound from surgery or trauma when the existing tissue cannot sustain a graft. Unlike a graft, which depends on developing new blood vessels, flaps rely on their own blood supply, which is surgically reconnected to nearby blood vessels.

**Figure 1:**
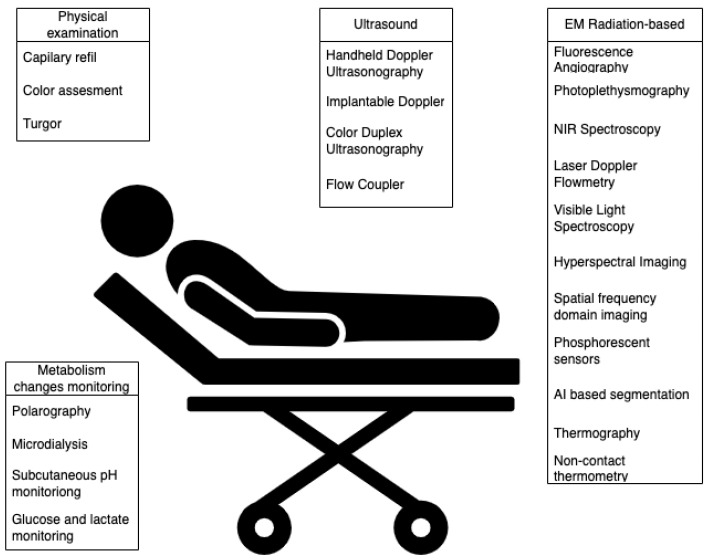
Overview of postoperative flap vitality monitoring techniques.

Even though, in recent years, autologous flaps have become an inseparable part of reconstructive plastic surgery, the risk of flap failure is still not negligible. Studies report success rates around 95% [1], which seems high, but we have to bear in mind that microvascular failure is a costly disaster and cannot be repeated indefinitely. Despite the significant progress in the development of monitoring techniques, major and minor complications, leading to the loss of the flap, still occur. In microsurgery and plastic surgery, it is crucial to recognize complications as soon as possible, which should enable the timely detection of micro-vascular failure and help ensure higher success rates of flap reconstructions. Despite numerous reviews describing postoperative flap monitoring techniques, no studies compare experimental and clinical methods from both a technical standpoint and clinical usefulness. Monitoring techniques were compared from a surgeon’s perspective (user of the monitoring system) and an engineer’s perspective (designer of the monitoring system). This thorough review discusses the multiple levels and aspects of state-of-the-art flap vitality monitoring systems and experimental solutions. As shown in Figure 1, we have assigned the methods to the following categories: physical examination, surface temperature measurement, ultrasound metabolism changes monitoring and light-based. In this article, we review all the methods presented. Furthermore, we presented accompanying challenges. In the first section of the review, we summarize fundamental flap classification based on movement mechanism, tissue type, and vascular supply. An angiosome concept delineating the safe clinical territory of flaps and perforasome theory were described. The most common postoperative complications were identified, analyzed, and presented. This knowledge is extremely useful in understanding the complex issue of monitoring tissue vitality.

Extensive searches using web search engines such as Google Scholar, PubMed, Research Gate, and IEEE Xplore were conducted. In researching existing methods, keywords such as, but not limited to “flap monitoring”, “tissue vitality”, TRAM monitoring, “flap care”, “postoperative monitoring”, “continuous monitoring”, “real-time monitoring”, “skin flaps”, “ischemia”, “vascularisation” have been used. Articles were organized, read, analyzed, and, if deemed valid, included in the review. Other reviews were excluded from citations after careful analysis. Appropriate papers were sourced from references of the reviewed articles. No generative AI tools were used to prepare this review.

## 2. Flap Characteristics

Fundamental classifications of flaps can be made based on the mechanism of movement, what types of tissues they include, or how their blood is supplied [2]. A concept known as the reconstructive ladder [3] dictates that the surgeon select the least complex variety to yield the desired result.

### 2.1. Movement Mechanism

There are three categories of flaps: local, regional, and distant. In the least complex method, local flaps are created by freeing a layer of tissue and then stretching it to refill an adjacent defect, i.e., covering the wound [4]. An advancement flap extends incisions parallel to the wound, creating a rectangle with one undamaged edge. This rectangle is then stretched to cover the wound, utilizing the blood supply from the uncut edge. Blood flow monitoring is usually unnecessary. Interpolation or regional flaps are positioned above or below healthy tissue to fill a defect while maintaining a blood supply connection. Examples include the latissimus dorsi (LD) flap, the pedicled transverse rectus abdominal muscle (TRAM) [5] flap for breast reconstruction, and the pectoralis major myocutaneous (PMMC) flap for head and neck deformities.

Distant flaps are the most complex, and are used when the donor site is far from the defect. Direct flaps, also known as tubed flaps, create a bridge between the recipient and donor sites, allowing the donor site to keep supplying blood while the recipient site forms a new blood supply. Free flaps have their blood supply cut off and then reattached to a new blood supply at the recipient site.

### 2.2. Tissue Type

**Figure 2:**
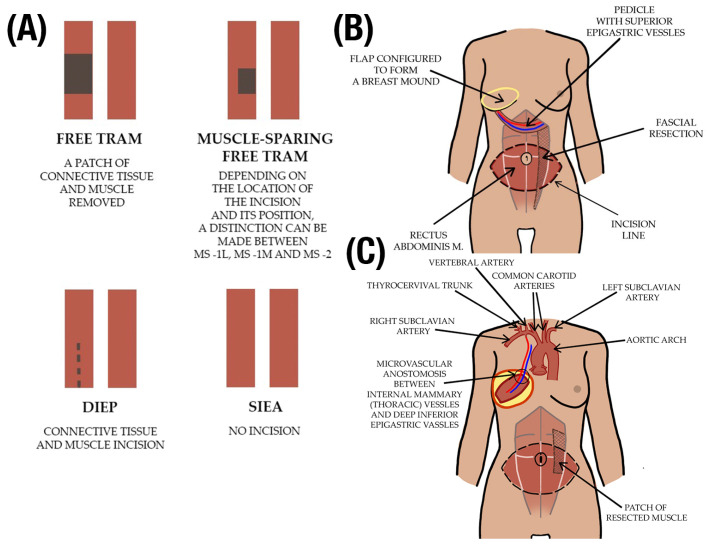
(**A**) A summary of free flap reconstruction methods comparing them by the amount of muscle involved is presented. Red blocks show an illustrative model of the transverse rectus abdominis muscle. Dark color represents amount of muscle removed (Free TRAM or MS-TRAM) or incised (in DIEP). (**B**) An example of pedicled flap: pedicled TRAM method employed in breast reconstruction surgery (**C**) An example of free flap: free TRAM method of breast reconstruction surgery.

The content of the tissue within them can also classify flaps. In Figure 2A, a summary of free TRAM breast reconstruction techniques, comparing them by the amount of muscle involved, is presented. Cutaneous flaps, which include the entire thickness of the skin, fat, and superficial fascia, are suited for small defect fillings and are generally supplied by a random blood supply, with examples including Z-plasty and DIEP flaps. Fasciocutaneous flaps incorporate deep fascia and subcutaneous tissue, suitable for larger defects and supported by a more robust blood supply, classified by the Cormack and Lamberty system [6], and include flaps like the lateral and posterior fasciocutaneous flaps, as well as the SIEA flap. Musculocutaneous flaps, comprising a layer of muscle tissue, address deeper deficiencies by adding mass and allowing for skin graft placement on top, with examples being the TRAM and latissimus dorsi flaps, among others. Flaps containing bone, like the fibula flap, are used for structural support in areas such as the jaw, while omental and intestinal flaps cater to specific reconstructions like chest wall deformities and restoring tubular systems, respectively.

### 2.3. Vascular Supply

The classification of flaps includes those based on blood supply. Axial flaps have a specific artery and vein supply, while random flaps lack a specific blood supply, and are instead supplied by the subdermal plexus. Axial flaps can be further categorized into reverse-flow flaps where blood is supplied by backward flow and pedicled flaps where the flap remains connected to the donor site by a pedicle carrying the blood supply. Random flaps lack a specific blood supply and rely on the subdermal plexus for blood flow. Pedicled flaps, like Pedicled TRAM (Figure 2B), remain connected to the donor site by a pedicle carrying the blood supply. In contrast, free flaps, like Free TRAM (Figure 2C), involve severing the vessels and anastomosing them to another blood supply.

### 2.4. Angiosome Concept

Ian Taylor’s concept of angiosome and choke vessels facilitated the task of delineating the safe clinical territory of flaps [7]. The angiosome is the area of skin and underlying tissues vascularized by the source artery. The angiosome theory divides the human body into three-dimensional tissue blocks delineated by specific arterial and venous supply [8]. Each angiosome defines a safe anatomical tissue boundary in each layer that can be transferred or joined together on the underlying source artery and vein as a tissue flap. Another essential part of Taylors’ paper is the description of types of connections between vessels called anastomoses. If the vessels have the same caliber, they create true anastomoses and maintain blood flow. On contrary, supply connection can be via choke anastomoses where the vascular outflow is a continuous system of arteries linked predominantly by reduced caliber vessels. Because, in this case, there is no pressure buffer effect, the blood is distributed via the branches of the artery up to some point. Then, it overflows into the branches of the adjacent vessels and escapes in a retrograde fashion via their main trunk, rather than perfusing a successive series of linked vascular territories [9]. An example of different types of TRAM flap in Figure 2 pictures this theory properly. If the blood supply from the donor side is based on the superior epigastric artery, as it is in pedicled TRAM, the primary portion of the skin island is in the second angiosome of this flap, receiving blood via choke vessels in the muscle. In conclusion, the blood supply in a pedicled TRAM flap is indirect. That is the reason why patients with a history of smoking, hypertension, obesity, chronic obstructive pulmonary disease, previous abdominal surgery, or even diabetes have been considered at high risk in terms of receiving a pedicled TRAM flap and achieving better results when operated with a free TRAM technique.

### 2.5. Perforasome Theory

The authors of the article [10] argue that while Taylor and Palmer have been influential in advancing our understanding of vascular anatomy through the angiosome concept, this theory is based on the vascular supply of source arteries. Nowadays, with the prominence of perforator flaps, the focus of vascular knowledge has shifted from the source artery to the perforator itself.

According to the Perforasome theory, a perforator possesses a specific vascular territory known as a perforasome. The vascular supply of perforators is quite intricate and follows some common principles. Both direct and indirect connecting vessels are crucial for the perfusion of perforator flaps. Depending on the size of the source artery, every perforator has the potential to become either a pedicle or a free perforator flap. A perforator flap based on a discrete perforator angiosome will reliably capture one adjacent perforasome across a choke anastomosis, or more if a true anastomosis exists [11]. This versatility allows for a wide range of perforator flap designs that can be customized to better address defects. Consequently, reconstructive surgeons now have more options for replacing tissue with similar tissue. The availability of clinically relevant perforators near the defect area limits flap design only for pedicle perforator flaps, making local flap alternatives more abundant.

Hyperperfusion of a single perforator can capture multiple adjacent perforasomes. The perforasome theory explains why large perforator flaps can be harvested based on a single perforator (e.g., extended anterolateral thigh flap [12]).

### 2.6. Most Common Postoperative Complications

#### 2.6.1. Vessel Thrombosis

**Figure 3:**
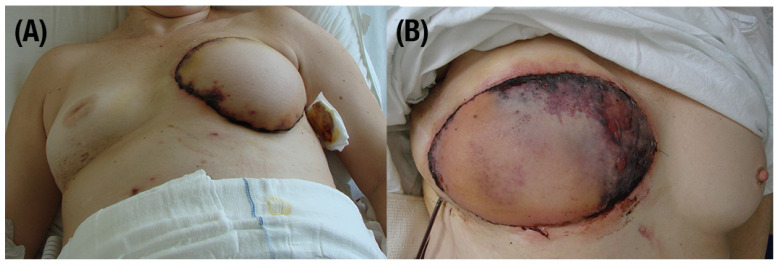
Complications after TRAM breast reconstruction. (**A**) Marginal ischemic necrosis. (**B**) Severe ischemic necrosis caused by vessel thrombosis.

Vessel thrombosis is a complication that can lead to serious consequences, such as necrosis (see Figure 3), which could result in flap loss. The blackening of the tissue indicates death of the cells. Vessel crisis not only can result in flap failure, but represents the most common complication in flap transplantation surgery. Among many reports, in free tissue transfer, venous thrombosisis occurs more often than arterial occlusion [13]. Chi Mao et al. show that the postoperative vessel thrombosis rate in flap transfers is 3.3%, 2.7% is venous thrombosis, and 0.6% is arterial thrombosis [14]. Causes of complications can be traced not only to surgeons’ skills, but also to patients’ medical history, including irradiation, smoking, infections, or vascular problems [15]. Thrombosis or venous stasis can be found as the most frequent cause of flap loss. In publications, venous thrombosis occupancy is 53.3%, followed by arterial thrombosis, with 46.7%. As numbers of publications show, venous thrombosis has a much higher rate of all complications [13,16,17,18]. Bianchi et al. show venous thrombosis as 5.1%, and arterial as 0.8% [19]. When vascular thrombosis is noticed, prompt surgical re-exploration should be performed to salvage the free flap. The time of rapid return to the operation room to manage the reestablishment of blood flow depends on the rate of successful salvage of an extended tissue [20]. Saving flaps from vessel thrombosis is highly linked with the time from incidence to detection. Transplanted tissue salvage rate drops from 62.2% to 21.4% if recognized after 16 h [21]. After 36 h from incidence, the chances become even lower [22].

#### 2.6.2. Arterial Vasospasm

Arterial vasospasm is a considerable pathological constriction that is usually found as a recalcitrant. Transferred flap tissue may exhibit restricted blood supply compared to non-transferred tissue, leading to partial necrosis of the reconstructed area. The solution is to expand vessels with the use of vasodilatation medicines, such as verapamil, nifedipine, calcium gene-related peptide, or lidocaine [23,24]. This type of treatment allows for improving blood flow, resulting in an increased survival rate [25]. The local regulatory mechanism, including pressure-related myogenic response, is most likely responsible for the resolution of blood flow. Comparing myogenic origin reaction and vasodilatation metabolites, the presence of arterial vasospasm cause immediately vasodilatation more probable with the first source [26,27]. Prolonged and moderate arterial vasospasm can lead to partial necrosis of the flap or even loss of transplant even when the flap is well vascularized.

#### 2.6.3. Hematoma

Hematoma is the second-most common reported complication in tissue transfer surgery. Its occurrence rate ranges between 0.2% and 9.1% [28]. The hematoma is caused by local bleeding. It leads to damage of the surrounding tissues as a result of an extrinsic pressure effect. The hematoma may also affect cellular and biochemical mechanisms. Cytokine-mediated inflammation, neutrophil infiltration, endothelial dysfunction, or pro-thrombotic shift can be disturbed, which through the production of reactive oxygen species can cause tissue injury with or without vascular occlusion [29]. Timely detection of the hematoma and its removal is very important for the survival of the flap, which may require re-operation [30]. The factors causing hematoma are debatable, but they include high intra-operative and postoperative blood pressure, preoperative smoking, incomplete tumor excision, or use of nonsteroidal anti-inflammatory drugs [31]. Although the risk of bleeding or hematoma formation can be reduced with the use of therapeutic anticoagulation, its routine usage is highly debatable [32].

#### 2.6.4. Inflammation

Inflammation is characterized by enlarged lymph nodes, firstly local, and increased inflammatory windowsills (such as C-reactive protein, Erythrocyte Sedimentation Rate, and procalcitonin). The management of postoperative infection depends on the current scale of this complication, as well as the patient’s general condition, comorbidities, adjuvant therapies, and disease progression. The basic and most important method of preventing peri-operative infections is asepsis, proper preparation of the operating field, and appropriate sterile clothing of the persons performing the procedure. The main task of asepsis is to remove germs. In order to protect the patient from infection, prophylactic antibiotic therapy is also used, the operating rooms are secured by using appropriate high-efficiency particulate air (HEPA) filters to ventilate the room or by additional sterilization with the use of ultraviolet lights.

### 2.7. Patient Monitoring Duration

When deciding on monitoring time, three key time windows for detecting flap failure are essential to improve the chances of salvaging a compromised flap. The most crucial monitoring period is within the first 48 h post-surgery, as this is when the flap is most vulnerable to issues such as vascular thrombosis, inadequate perfusion, or mechanical complications. Clinical research underscores that the majority of flap failures, resulting from both venous and arterial thrombosis, occur within this initial timeframe. The majority of flap failures occur within the first 48 h [33,34]. According to the review of 109 studies featuring 44,031 free flaps [35], the first 48 h following surgery are when close flap monitoring is most beneficial, since it allows for quicker identification of vascular compromise, early salvage, and better results.

Many reports suggest even longer monitoring period [18,36,37,38]. The period up to 72 h post-surgery, although with a decreased risk, remains an important window for monitoring, given that delayed complications can still arise. In the study of Kroll et al., 95% of circulation compromise cases took place in the first 72 h after operation [39]. In some publications, we can find information that even the first 72 h period may be ineffective in successful salvage [17]. Beyond the 72 h mark, whilst the bulk of flap failures have typically been identified, observation should be extended up to a week post-surgery. This duration allows for the detection of late-onset complications which, albeit rarer, can threaten the viability of the flap. Continuous clinical assessments, in tandem with advanced monitoring technologies, offer a thorough approach to identifying even the most subtle changes in perfusion or the condition of the flap, ensuring the best possible outcomes for patients post-reconstruction. Hirigoyen et al. [40] show that 50% of postoperative observations lasted 48–72 h period, 33% of surgeons monitored their flaps longer than 72 h, and the window of 24–48 h was performed in 16%.

### 2.8. Flap Failure Detection Time Window

Protocols for flap monitoring between plastic surgery units vary widely. A common regimen involves care every 30 min for the first 3 h, every hour for up to 48 h after surgery, and every 2 h for up to 72 h after surgery. After that, care should be provided every 4 to 8 h until the patient is discharged from the hospital [41]. Free flap monitoring: in a review of current practice, Hirigoyen et al. [40] have shown 75% of surgeons conduct observations at intervals of three hours or less, 23% chose a period of 3 to 6 h between the monitoring, while only 2% felt convenient with an interval of more than 6 h. According to large study by Patel et al. [1], reduced resident monitoring frequency does not alter flap salvage nor flap outcome. When the monitoring frequency was analyzed, flap compromise was observed in 12% of flaps monitored every 4 h, 8% monitored every 8 h, and 6% monitored every 12 h. The overall success rate for the flaps was 92% when monitored every 4 h, 93% when monitored every 8 h, and 95% when monitored every 12 h. In the study by Vijan and Tran [42], the mean time of successful salvage and failure was 127 min and 192 min, respectively (the latest flap they were able to salvage was 188 min after detection of thrombosis).

## 3. Monitoring Techniques

The surgeon performing tissue transplantation, cannot truly evaluate the procedure as successful until the flap has survived the first few days. Postoperative observations start in the operating room (OR), immediately after the release of the microvascular clamps. Frequent clinical examination is the gold standard in the monitoring of microsurgery reconstructive transplantation [43]. Postoperative monitoring is irreplaceable in achieving a high rate of success in tissue transfer [13]. Among vast variety of techniques that can be found in the literature, there is no one method that is universally effective. Ideal flap monitoring techniques should be reliable, reproducible, easy to interpret, non-invasive, safe, inexpensive, and simple to use. They should also differentiate vessel spasm from occlusion, and venous complication from arterial. Nowadays, there is no such method that fulfills all of the requirements above. According to a retrospective cohort study comparing two monitoring protocols [44], free flap outcomes did not vary based on resident physicians’ degree of flap monitoring. Moreover, the data presented support the ability of a high-volume, well-trained, nursing-led flap monitoring program to detect flap compromise efficiently while limiting resident physician obligations in the age of resident duty hour restrictions.

### 3.1. Physical Examination

Differences between an arterial and a vein occlusion are presented in Table 1.

#### 3.1.1. Capillary Refill

By assessing the time of capillary refill due to pressure, it is possible to assess the effectiveness of the blood supply to the flap. With the use of, for example, a spatula, the place is temporarily pinched; when it is lifted, the tested surface should be pale, and the return to the previous color should occur within 1–3 s. This is a sign of a balanced flow and drainage of blood. If this time is longer, there is insufficient arterial flow and it usually takes more than 3 s, usually more than 5, to refill the capillaries. However, shortening the inflow time to less than 1 s, or the complete lack of color change, indicates impaired venous outflow.

#### 3.1.2. Color Assessment

The color assessment of a flap can help evaluate circulatory disturbances. A scale of 1 to 10 is used, with 1 being white or pale, 5 being pink, and 10 being dark pink or black [17]. Different methods like the Munsell skin tone chart or Fitzpatrick’s skin types are used to assess this. Arterial disorders cause the flap to turn pale, while venous disorders cause swelling and purple pigmentation. Venous disorders instead can be manifested by swelling of the affected area and the appearance of purple pigmentation. The changes do not have to involve the entire surface of the flap, and may be present just locally. In more darkly pigmented skin, erythema is inapparent, and is replaced by patches of darkened skin color. In the early stages of complications, the changes in skin tone may be difficult to notice or not present at all. This is the reason why other methods of flap assessment are also recommended.

#### 3.1.3. Turgor

An excessively hard-to-touch flap may indicate an excessive presence of venous blood. Touching the flap does not give the impression of softness but resistance. Swelling of the flap is a less useful measure of blood flow assessment and can only be detected at a later date.

### 3.2. Biochemical Methods

#### 3.2.1. Polarography

Heyrovsky established that applying a correct voltage to a mercury electrode produced a current proportional to the electrolyzed material tension in the surrounding liquid (i.e., oxygen (O_2_) in Transcutaneous oxygen pressure (TcPO_2_) [45]). He was awarded a Nobel Prize in recognition of his work.

Tissue Oxygen Tension Measurement (PTO_2_) is the directly measured local partial pressure of oxygen in a specific tissue. A method of PTO_2_ determination in man by equilibration through intact skin was first proposed by Baumberger and Goddfriend [46]. A novel polarographic electrode in which the platinum cathode was covered with a semipermeable membrane to produce a more uniform O_2_ layer was proposed by Leland Clark, Jr. [47]. Huch et al. [48] described the use of heated Clark electrodes as a feasible approach for monitoring skin surface PTO_2_. The method was first used clinically to monitor oxygen levels in newborns during and after delivery. The authors of [49] used an implanted Silastic catheter to monitor Tissue Oxygen Tension in Postoperative Patients. In this technique, an oxygen-permeable catheter was introduced via either a cannula or a surgical wound. To measure PO_2_, hypoxic saline with PO_2_ in the range of 5 to 10 mmHg, obtained by bubbling nitrogen gas through normal saline, was introduced into a tube. Five glass capillary tubes were filled and then measured.Kamolz et al. [50] described using oxygen partial pressure monitoring in early recognition of flap failure. The Licox^®^ Catheter PTO_2_ Micro-Probe instrument is used for continuous determination of oxygen partial pressure in body fluids and tissue. Wechselberger et al. [51] indicated that the probe’s distance to the pedicle/vessels within the flap is one of the most significant limitations of this method. Jonas et al. [52] pointed out high sensitivity to micromovements of the probe inside the flap to the regulatory changes in the flap microcirculation. Raittinen et al. [53] rated the Licox probe as a sensitive and reliable tool for monitoring microvascular reconstructions in the head and neck area. The device has recognized all the patients who needed re-operation with a sensitivity of 100% and specificity of 88%. The overall success rate was 99.2% (117 of 118), and the flap salvage rate was 88% (seven of eight), respectively. On the other hand, in comparison between two cohorts of patients, Arnevz et al. [54] evaluated the Licox system for monitoring buried free flaps. According to their findings, PTO_2_ monitoring should only be used as a supplement to other systems. Furthermore, its use, compared to near-infrared spectroscopy or clinical bedside monitoring, was not found to be cost-efficient.

In a preliminary report [55], the usefulness of an oxygen partial tension monitoring system for postoperative flap monitoring has been evaluated. The authors demonstrated that PTO_2_ measurements predictably indicate flap failure when lower than 9 mmHg, or survival when higher. Alarmingly low values can prompt immediate re-operation, increasing the chances of flap salvage. The authors, however, indicated that the main limitation is the high cost. They could however be reduced in the long term if the device was used regularly. Further clinical trials have been recommended in order to confirm the results, and better assess the role of such technique in flap monitoring.

The transcutaneous pressure of carbon dioxide (TcPCO_2_) method, first introduced by Severinghaus [56], utilizes the arterialization of cutaneous capillaries by applying heat. A glass pH electrode, and the reference silver/silver chloride electrode, a heating element, a temperature element, and an electrolyte reservoir make up the sensor. When applied to the skin, heat causes the cutaneous capillaries to dilate and increases the skin’s permeability to CO_2_. Diffusing through the membrane, CO_2_ combines with water to generate carbonic acid, which splits into bicarbonate and hydrogen ions. The pH changes resulting in a potential differential between the two electrodes. Under stable hemodynamic settings, it has been demonstrated that the final recording accurately represents the pressure of ${\mathrm{CO}}_{2}$ in the cutaneous capillaries, which correlates well with level partial pressure of carbon dioxide obtained from arterial blood (between 20 and 74 mmHg) [57].

In sensors with an electrochemical PTO_2_ (Clark-type) and tcPCO_2_ measurement, the Clark-type electrode and its inherent oxygen consumption influence pH within the diffusion chamber. A novel transcutaneous OxiVenT Sensor combining optical PTO_2_ and electrochemical TcPCO_2_ monitoring with reflectance pulse oximetry has been evaluated by van Weteringen et al. [58]. According to their findings, OxiVenT Sensor is clinically usable and provides good overall accuracy and negligible TcPO_2_ drift. The proposed solution has not yet been clinically evaluated in flap vitality monitoring.

#### 3.2.2. Microdialysis

Microdialysis is an experimental invasive, non-continuous, and indirect monitoring method proposed by Delgado et al. [59] for neurotransmitters and ischemia detection in monkeys. This technique places a microdialysis catheter or probe (with an open needle) into the tissue. Later, it is connected to a small pump, which infuses physiologic fluid into the dialysis membrane.

Aliquots of the perfusate can be analyzed on the tissue content of glucose, lactate, pyruvate, and glycerol metabolite concentrate. A rising lactate-to-pyruvate ratio and falling glucose indicate arterial compromise [60]. Tissue lactate and glucose concentrations, particularly their relationship (the lactate-to-glucose ratio), can indicate ischemia and help choose the decision to perform a second procedure. Complete or partial flap necrosis may also be predicted by a high lactate-to-glucose ratio [61]. A rising glycerol level indicates cell membrane damage and may indicate venous congestion and arterial compromise [62].

Microdialysis allows for early detection of vascular compromise even before any clinical signs [63]. According to the authors of [64], microdialysis provides excellent diagnostic accuracy and enables the early detection of ischemia in postoperative flap monitoring. In a comparative study, microdialysis values proved flap viability sooner than external Doppler ultrasonography, making it an excellent tool for postoperative monitoring. With the appropriate thresholds for glucose and lactate concentrations and glucose/lactate ratio used as a new parameter, it can help potentially avoid unnecessary re-explorations and reduce flap ischemia times [65].

On the downside, the procedure takes around 30 min, and a learning curve is required to optimize the system. Although the positive predictive value in most studies oscillates around 90%, showing cases going as low as 22% [66] can be found. The authors, however, underline that a learning curve may have caused it. A negative predictive value is reported to be as high as 100%. Other researchers, such as Morouzis et al. [67] and Dakpe et al. [68], also explored the feasibility of microdialysis in flap monitoring. Microdialysis is a costly method, with an analyzer itself costing over USD 50,000 and catheters being disposable.

#### 3.2.3. Subcutaneous Tissue pH Monitoring

An experimental, invasive, and continuous monitoring technique was first proposed by Dickson and Sharpe [69]. After initial calibration, a glass pH probe was placed in a small stab incision, penetrating tissue at a depth of around 3 mm and connected to a recording monitor with a coaxial cable. The technique allows for detecting vessel occlusions with a response time of 30 s to 1 min. It has been shown that it is possible to differentiate both arterial and venous occlusions by their pH curve. Although the results presented in the article were auspicious, and the authors recommended further exploration in this field, no other studies, including pH probes in flap monitoring, were found.

#### 3.2.4. Glucose and Lactate Monitors

According to [70], hypoxia causes increasing lactate values and decreasing blood glucose simultaneously during ischemia, suggesting that such measurements could help evaluate flap blood flow. A recent study suggests that flap capillary lactate measurement is an easy, quick, cost-effective, and objective tool for checking the viability of flaps [71]. In article [72], researchers employed a simple glucose monitor for diabetic patients (Medisafe mini GR-102, Terumo, Japan). Obtained results indicated that flap-glucose concentration could be one of the valuable tools in flap monitoring. In a 2018 case report, Tachi et al. [73] proposed using a continuous tissue glucose monitoring device (CTGMD) in postoperative flap monitoring. Tissue glucose levels in the flap were monitored by a CTGMD (Freestyle Libre^®^, Abbott, Green Oaks, IL, USA), and blood glucose levels were observed in parallel by conventional sampling of the flap.

In [74], a simple measurement procedure was proposed. Postoperative measurements were obtained at the time of wound closure and 2, 4, 8, 12, 16, 20, 24, 36, and 48 h after wound closure. The flap was pricked with a 25-gauge LS Lancet, and a small amount of blood was extracted. A handheld lactate monitor (Arkray Lactate Pro 2) and glucose monitors (Medisafe and Arkray) were employed. The study showed that lactate value, rather than blood glucose value, statistically significantly detected blood flow obstruction.

In [75], researchers investigated trends in interstitial fluid glucose concentration (IFG) in flap monitoring by measuring IFG automatically and continuously. Results indicate that IFG may complement conventional flap monitoring, particularly in the early postoperative period. In [76], Colman et al. suggest that the pinprick glucose test is an easy, reliable, accessible method that can be performed by non-medical personnel.

### 3.3. Ultrasound-Based Methods

#### 3.3.1. Handheld Doppler Ultrasonography

In combination with physical examination, Acoustic Doppler Ultrasonography is one of the most often employed methods of flap monitoring. A monitoring device based on a Doppler effect—a phenomenon of changing the frequency of the ultrasound wave due to contact with moving blood—is cheap, simple, easy to maintain, and allows for non-invasive measurements. In its simplest form, the Handheld Acoustic Doppler Ultrasonography generates a sound appropriate to the blood flow volume. Also, more sophisticated devices plotting measurement results, such as Hadeco DVM-4500, can be used. During surgery, the operator often marks the place closest to the perforator with a suture. There are a few drawbacks to this technique. Acoustic Doppler measurements are relatively time-consuming. The method does not allow for continuous measurements. One of the most significant drawbacks is that its precision may vary between operators, and since it is a handheld device, results are subjective; thus, it is almost impossible to notice initial changes in blood flow. Also, the method is characterized by low specificity since it is easy to overlook smaller vessels. A new design of handheld Doppler shaft for postoperative monitoring has been recently proposed [77]. It can easily be inserted in narrow, limited spaces like the oral cavity.

#### 3.3.2. Color Duplex Ultrasonography

This method, like Acoustic Doppler, employs ultrasound to directly visualize vessels and blood flow in them using the Doppler effect. It is more sophisticated, since it allows one to monitor the direction of a blood flow and plot it on a screen in real-time. Higher frequency transducers enable operators to measure at superficial depths with the ability to detect vessels as small as 0.2 mm [78]. Similarly to an Acoustic Doppler, Color Duplex Ultrasonographs specificity is relatively low and varies from an operator. The device is much more expensive, has to be operated by a radiologist, and requires technical input from a performing surgeon. For the above reasons, Color Duplex Ultrasonography is not frequently used in routine post-operative monitoring.

With a price from USD 30,000 up to over USD 225,000, a Color Duplex Ultrasonograph is considered a costly yet easily accessible monitoring device. This monitoring technique is far from ideal for flap monitoring. It is non-continuous, and its usage requires trained personnel.

#### 3.3.3. Flow Coupler

Often, venous anastomosis is created using couplers–hollow polyethene rings on which vessel ends are mounted and clamped, ensuring a tight seal. Company Synovis has introduced a flow coupler equipped with a Doppler probe. After anastomosis, wires are led via the surgical wound and connected to a power and sound source. The monitoring device generates auditory output corresponding to blood flow changes. Measurements are continuous, which may lead to earlier OR takebacks in cases complicated by venous thrombosis. After the postoperative period, the probe is simply pulled out from the patient’s body, leaving the coupler in its place. Patient movement may, however, cause accidental removal of the probe. Studies have shown that this method is more sensitive to identifying vascular compromise than implantable arterial Doppler. There is, however, an elevated false positive rate, which necessitates further physical exam [79].

#### 3.3.4. Implantable Doppler (ID)

ID utilizes the Doppler effect similarly to a flow coupler, but unlike it, ID may be used at both arterial and venous anastomoses. A probe is typically wrapped around the site of anastomosis. The signal is transmitted through a wire brought out of the surgical incision and connected to a sound and power source. Similarly, the flow couplers monitoring device generates auditory output corresponding to blood flow changes. ID provides continuous measurement, since implantation and can detect subclinical vascular compromise without delay, resulting in a higher flap salvage rate. The device can immediately detect compromised arterial blood flow. However, an arterial Doppler signal will persist for several hours after venous thrombosis. In the case of probe implantation around veins, a compromised arterial blood flow can be detected almost immediately. Venous probes allow detection of venous occlusions immediately and arterial occlusions with a mean time of 6 ± 2.4 min. Arterial probes enable the detection of arterial occlusions immediately, but the detection time of venous occlusions is delayed by 220 ± 40 min [80]. Therefore, probe placement around the vein provides a marked advantage compared to a probe placed around the artery and also allows for arterial blood flow monitoring [81]. According to a recent study, implantable Dopplers are an effective method for evaluating the postoperative success of completely buried free flaps [82]. Compared to Single Photon Emission Computed Tomography (SPECT), the implantable Doppler revealed sufficient specificity in monitoring buried fibular graft. However, SPECT showed a lower sensitivity than the implantable Doppler method [83].

The disadvantages of implantable Doppler include endothelial trauma during device application and vessel twisting. It is also worth noting that patient movement may cause probe displacement. This can alter the signal even in the setting of standard anastomosis. After the monitoring period, the probe is removed by pulling it out of the patient’s body. It has been proven that ID exhibits fewer false positives than venous Doppler [84]. Using a Cook–Swartz implantable Doppler probe is associated with a cost of about USD 300 [81]. The authors of [85] deemed the Cook–Swartz Doppler probe a helpful adjunct to clinical monitoring but underlined complications such as one retained wire, one pedicle laceration during extraction, and one clot around the probe interrupting signal.

### 3.4. Electromagnetic (EM) Radiation-Based Methods

**Figure 4:**
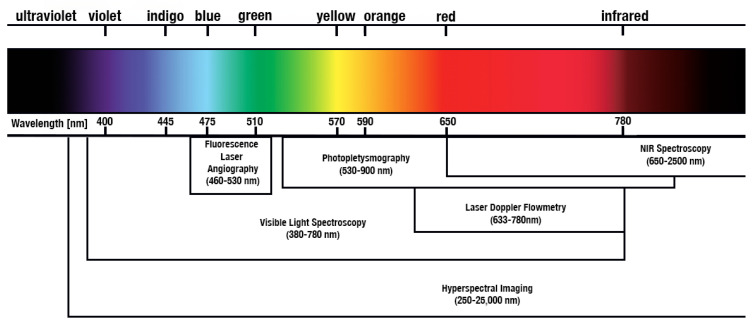
Electromagnetic (EM) radiation-based methods classification based on the wavelength.

In Figure 4, different radiation-based methods were classified based on the observed spectra.

#### 3.4.1. Fluorescence Angiography (FA)

FA is a visual blood flow monitoring technique in which a patient is injected intravenously with indocyanine green dye (ICG). Blood flow can be observed in real-time with a device consisting of a laser light source and near-infrared camera. Arterial inflow, venous return, and tissue perfusion can be visualized during and post-operative periods. ICG molecules strongly bind to plasma proteins, but exhibit a short plasma half-life of 3–5 min. This allows repeated injections without ever reaching toxic levels [86]. Moreover, in contrast to the continuous methods, ICG-FA only illuminates the flap perfusion area within a specific time range.

The authors of the 2002 article [87] indicated that FA may be a proper supplementary technique for intraoperative monitoring of free flaps. Despite not achieving all of the criteria for an ideal monitoring device, ICG imaging appears to be a valuable tool for detecting impaired microcirculation. According to the authors, it provides a convenient, quick, and inexpensive measuring technique for the monitoring staff. With proper future development and adequate experience with the technology, its clinical applicability is unquestionably justified.

Krishan et al. [88] indicated numerous restrictions associated with FA. According to the study, the method does not help predict or objectively assess venous congestion of flaps; moreover, the technique cannot map buried flaps, such as in pure muscle.

In [89], ICG FA has proven feasible and safe for detecting flap vascular problems intra and postoperatively. ICG FA slope and amplitude were abnormal in patients with flap vascular problems before clinical examination. The authors, however, pointed out that preliminary clinical trials had too few patients and complications to give statistical proof of FA ICG effectiveness.

Adelsberger et al. [90] evaluated the effect and applicability of indocyanine green FA in bedside monitoring of free flaps. In the study, ICG FA was used in parallel with the clinical examination of free flaps. According to the findings, this conjunction can help correctly identify vascular thromboses and decide if revision surgery is required in cases of perfusion impairment that are not readily apparent.

Article [91] presents a prototype of a handheld ICG Near-Infrared Fluorescence Imaging Device based on a modified action cam with an additional lens and band-pass filter. In [92], ICG FA was portrayed as a standard clinical tool to forecast flap failure following microvascular repair in the head and neck region. According to the authors, ICG FA might eventually offer an unbiased way to determine intraoperatively whether or not to make an urgent revision to an anastomosis to avoid flap loss or salvage surgery. Although FA provides a high sensitivity in detecting well-perfused and non-perfused tissue [93], ICG dye is relatively expensive, which makes this method quite inefficient in terms of cost, especially during long-term monitoring.

According to the study’s authors, assessment of flap perfusion via fluorescent angiography during initial flap harvest or when flap compromise is suspected postoperatively can guide decision-making in free flap reconstruction of the head and neck and can be added to existing planning and management paradigms [94].

#### 3.4.2. Photoplethysmography

A total of 97% of the oxygen present in the blood is bound to hemoglobin in red blood cells, and the remaining 3% is dissolved in plasma and cells. Oxygen saturation is the ratio of oxygen-saturated hemoglobin to total hemoglobin in the blood. Under normal conditions, the oxygen saturation is close to 100%, and the range up to 97% means good gas exchange. We observe hypoxia, i.e., the lack of oxygen in tissues in relation to the demand, leading to hypoxia in the body when the ${\mathrm{SpO}}_{2}$ level drops below 90%. Light is transmitted, absorbed, or reflected when emitted onto the tissue. The relative absorption or reflection of light of different wavelengths is used in many monitoring methods. This type of measurement is called spectrophotometry and is based on the Beer–Lambert law. The optical spectrum of infrared light uses an optical window of 700–900 nm, in which the light easily penetrates the skin, tissues, and also bones. Within this range, hemoglobin and deoxyhemoglobin are strong light absorbers. The differences in their absorption spectra make it possible to measure the relative changes in hemoglobin concentration using the light attenuation factor of different wavelengths. To assess oxygen saturation, at least two waves with frequencies distant from the isosbestic point of 810 nm should be selected; one with a lower frequency and the other with a higher frequency. Brinkman and Zijlstra originally described the non-invasive measurement of arterial hemoglobin oxygen saturation (Sa02) using skin reflectance spectrophotometry in [95]. According to the authors of [96], Green Light Photoplethysmography has provided a precise and responsive method for determining venous and arterial compromised flaps almost immediately after the onset of ischemia. A three-wavelength reflective optical sensor and processing system based on photoplethysmography (PPG) principles has been developed to investigate blood volumetric changes and estimate free flap blood oxygen saturation continuously and non-invasively in DIEP free flaps during the postoperative period. The technique was tested on 15 patients undergoing breast reconstruction surgery with a DIEP-free flap [97].

#### 3.4.3. NIR Spectroscopy (NIRS)

NIRS is a noninvasive optical sensing technique that uses light within the red and near-infrared (650–2500 nm) range to assess changes in an object’s optical properties. In 1976, the National Institutes of Health (NIH) issued Prof. Frans F. Jöbsis for exploratory research applications in physiology or medicine focused on novel techniques [98]. Jöbsis presented his findings the article [99].

NIRS in postoperative monitoring of tissue flaps is described in [100]. A. Repez and the research team monitored 48 patients after breast reconstruction. In total, 13 cases of anastomotic thrombosis (2 arterial and 11 venous) occurred in 10 patients. In each case, NIRS made it possible to detect flow disturbances before the appearance of visible changes. There were also no false alarms. Rapid detection enabled the salvage of 70% of the transplants. The survival rate of the flaps reached 94%.

Most NIRS investigations use continuous-wave NIRS (CW-NIRS), which can only provide information on relative changes in chromophore concentrations, such as oxygenated and deoxygenated hemoglobin, and estimations of tissue oxygen saturation. Another type of NIRS, frequency-domain NIRS (FD-NIRS), has several advantages: it can directly measure optical path length, thereby quantifying the scattering and absorption coefficients of sampled tissues and providing direct measurements of absolute chromophore concentrations.

The authors of the recent article [101] conclude that NIRS holds promise as an objective and valid method for clinical evaluation of buried bone flaps. A wide variety of NIRS devices for measuring tissue oxygen saturation are commercially available [102].

In 2009, the StO_2_ Tissue Oxygenation Monitor cost around USD 16,500, and the disposable sensor was priced at USD 150 [103]. Most commercially available NIRS probes are disposable. For example, the cost of the ViOptix probe in 2019 was as high as USD 1000 [104], making the method less attractive. Even though clinical evaluation in conjunction with Vioptix is more effective for flap monitoring following autologous free flap breast reconstruction, clinical examination alone is the most cost-effective flap monitoring alternative [105].

Guo et al. [106] reported a wireless, miniaturized, minimally invasive near-infrared spectroscopic system designed for uninterrupted monitoring of local-tissue oxygenation. A skin-mounted NIRS device (ViOptix) has been used as a reference. In vitro tests and in vivo studies in porcine flap and kidney models demonstrated the ability of the developed system to measure oxygenation with high accuracy and sensitivity continuously. The sub-mm form factor of this device enables minimally invasive use in deep tissue, making it ideal for monitoring the buried flaps.

According to [107], the most critical aspects of monitoring the flaps’ microvascular perfusion and vitality utilizing NIRS lie in its function of recording the dynamics of changes in the values of absolute oxygen saturation, alongside detecting a 30% decrease in tissue saturation over a 60-minute interval before the clinical changes in the microvascular flap become observable.

The authors of [108] proposed a protocol for monitoring free flaps with skin paddle using the combination of the NIRS and ultrasonography, a noninvasive and reliable method for the early identification of postoperative complications. This protocol provides continuous and objective information (NIRS) and specific hemodynamic and anatomical details of the vascular status of the flap (ultrasound), and it improves the overall salvage rate (25% vs. 72.7%, *p* 0.039) and reduces the need for specific staff with continuous on-site presence for flap monitoring.

In a recent study [109], researchers proposed the use of a continuous noninvasive near-infrared spectroscopy sensor on the head and neck free flap with a second sensor on non-operated tissue to improve the distinction between systemic hypoperfusion and flap compromise. The second sensor may enhance the distinction between flap compromise events and systemic hypoperfusion.

#### 3.4.4. Laser Doppler Flowmetry (LDF)

LDF is a non-invasive method of continuously measuring microcirculation perfusion in various tissues. Blood flow is estimated by irradiating the tissue with coherent light at a specified frequency. The light reflected from the erythrocytes is then measured and processed in the frequency domain. A helium-neon (HeNE) laser with a 633 to 780 nm wavelength is used as the light source. The test results are presented as erythrocyte flow values expressed in relative perfusion units (PU) due to the inability to calibrate the measurement to physiological units. Other output parameters include flux, velocity, and electron concentration. The Laser Doppler flowmetry method has been used in the non-invasive study of local flow within the skin and oral cavity, as well as in the invasive, intraoperative assessment of internal organs such as the heart, brain, kidneys, bones, and intestines [110]. LDF is also used to assess blood flow in transplanted tissues and dental pulp after injuries. The biggest problems associated with using LDF include susceptibility to the subject’s movement—artifacts created during the test can completely change its result.

The authors of [111] suggest the capability of early detection of perfusion incompetency. According to their findings, LDI may be recommended as an additional post-operative monitoring device for free muscle flaps, for early detection of suspected failing flaps and validation of other methods. Systemic diseases such as hypertension and diabetes and local inflammatory changes may also influence the assessment [112]. While there are no standardized criteria for diagnosing blood flow restriction, many research groups have suggested that values greater than 2.0 LDF demonstrate flap viability. However, these values may vary depending on the type of transplant, device model, and sensor placement. Two extensive studies with a total population of 326 cases showed the ability to detect blood supply disorders at the level of 94%, not including cases where the electrodes had to be reattached [113,114]. The cost of a Perimed Periflux 5000 monitor in 2020 was estimated at around USD 35,000. In the 2019 comparison of LD with NIRS, the authors of [104] indicated that average sterilization cycle per probe was 11, and 1 broke after 14 cycles. The cost per flap for this LD probe was USD 154. The main limitations of LDS are shallow penetration depth, relatively high monitoring cost and susceptibility to patient movement.

The O_2_C (oxygen to see) developed by LEA Medizintechnik combines a laser Doppler flowmeter and a tissue spectrometer. This study showed that attached surface probes are comparable to the standard unattached surface probes used with the O_2_C analysis system for flap perfusion monitoring regarding technical feasibility and patient safety. However, cut-off values for attached surface probes that indicate sufficient flap perfusion need to be determined to maintain the reliability and usefulness of the O_2_C analysis system as a method for flap perfusion monitoring [115].

#### 3.4.5. Visible Light Spectroscopy (VLS)

VLS oximetry uses shallow penetrating visible light to measure hemoglobin oxygen saturation (StO_2_) in small tissue volumes [116]. VLS has been proven to accurately monitor tissue perfusion in numerous animal models and identify ischemia during vascular surgery, tumor ablation, and gastrointestinal endoscopy [117,118].

A 2011 article [119] portrayed Visible light spectroscopy as a novel technique for predicting ischemia in microvascular cutaneous soft tissue-free flaps. Twelve patients were observed post-free flap reconstructions. Changes in StO_2_ and total hemoglobin concentration (THB) were recorded continuously for 72 h after the reconstruction in all flaps. VLS appeared to effectively predict early ischemia problems following free flap reconstructions.

In the 2013 publication [120], a monitoring device (T-stat) utilizing white light spectroscopy was compared with traditional flap monitoring techniques, including pencil Doppler and clinical examination. The T-stat system detected a rapid fall in saturation from around 50% to less than 30% 50 min earlier than it was possible using the physical examination approach. The estimated cost of a T-Stat monitor is USD 25,000, with each probe costing USD 100.

In [121], visible light absorption in diffuse reflectance spectroscopy for non-invasive, continuous, real-time monitoring of flaps sensor was proposed. The results support that the proposed approach has great potential, not only for fast detection of ischemia, but also for clear differentiation between arterial occlusion and venous occlusion.

#### 3.4.6. Hyperspectral Imaging (HSI)

Hyperspectral imaging is a continuous, non-invasive, in vivo imaging technique that incorporates spectroscopy concepts with contact-less imaging to offer information about morphology, physiology, and tissue composition. HSI has been well characterized in a 2014 review article by Lu and Fei [122]. HSI has been employed for perfusion monitoring of transplanted flaps for the covering of severe non-healing wounds in plastic surgery, as well as for perfusion measurements of chronic wounds and in other experimental areas of use [123,124].

Kulcke et al. reported about a new compact hyperspectral camera system designed for use in clinical practice [125]. In 2021, two independent research teams compared HSI and clinical monitoring techniques in terms of their ability for early detection of impaired free flap perfusion [126,127]. The authors of both publications indicate that the technique may detect complications at a very early stage when clinical. Recent methods have effectively performed automatic tissue categorization and differentiation ex and in vivo based on hyperspectral cubes utilizing deep learning algorithms (neural networks and computer vision) [128,129,130]. Hyperspectral Imaging exhibits dependence on ambient illumination, especially in intraoral applications. The issue of applicability in heavily pigmented individuals due to extended light absorption also arises.

#### 3.4.7. Surface Temperature Measurement

Temperature measurement can also be used to assess circulatory disturbances. To make this assessment, it is necessary to compare the temperature of the transferred flap on its surface and the temperature of the adjacent skin as the test sample. The free tissue flap where blood usually flows should have a temperature close to the surrounding area. It is assumed that differences more significant than 1–3 °C may indicate problems with blood supply [131]. Various measurement methods can be used to determine the differences in the temperature of the transplant. A simple liquid crystal temperature probe may be placed on the flap tissue, with a second placed on the adjacent normal skin. This method is less effective on buried flaps and intramural free flaps.

The last few years have seen rapid development of non-contact temperature measurement methods. Two main techniques dominate point measurement: non-contact infrared thermometers (NCITs) and measuring temperature over a larger area using infrared thermographic cameras (IRT). It has been proven that NCITs and IRT can provide accurate and reliable body temperature measurements in specific settings and conditions [132]. If for diagnostic purposes or to monitor the condition of tissues, organs, or the entire body, measuring the temperature at one point is enough, digital thermometry is a cheap and sufficient solution. Advanced measurement systems can be used, such as Perimed PF 5020—temperature unit used to perform local heat provocation and temperature measurements or simple non-contact thermometers like BRAUN NTF3000 [133]. The main disadvantage of such a measurement is the fact that the temperature is determined for a small (almost point) area. This may introduce measurement errors, e.g., overestimating the temperature if we hit an artery that supplied blood to the surface or underestimating the temperature in a very hairy area. For spot measurement, to ensure more excellent reliability, the measurement should be repeated several times which, in turn, lengthens the entire procedure.

#### 3.4.8. Thermography

Thermography is a non-invasive technique for diagnosing physiological changes by measuring body temperature. It was first used clinically in 1956, showing that neoplastic breast tissue has a higher temperature than healthy tissue [134]. Initially, the medical community enthusiastically adopted thermography, but due to diagnostic errors and imperfect equipment, it later faced skepticism following numerous unnecessary surgeries for falsely diagnosed cancer. Nowadays, advances like Dynamic Infrared Thermography (DIRT) and Active Dynamic Thermography (ADT) are improving non-contact perfusion measurements in breast reconstruction [135,136,137]. Thermograms are graphical representations of emitted, transmitted, and reflected infrared energy. However, accurately determining the temperature of an object is challenging due to multiple infrared sources. Modern thermal cameras address this with algorithms that adjust for environmental noise, providing high thermal and geometric resolution. Typically, cameras can record up to 50 images per second, while high-end solutions allow for capturing up to 1000 frames per second [138,139]. Active Dynamic Thermography maps temperature changes over time in response to external stimuli, offering insights into an object’s thermal properties based on its heating or cooling response. The aim of the test is to determine the thermal response of the object during the transient processes. The properties of the object may vary depending on the type of stimulation—thermal (external) or pharmacological (internal). The response is in the form of temperature change, and the rate of change contains information about the values of heat capacity and thermal conductivity.

The Pennes bio-heat transfer equation, first introduced in [140], is given as:

$$\rho c_{p}\frac{\partial T}{\partial t}=\nabla \cdot \left(k\nabla T\right)+Q_{b}-\rho c_{b}\omega_{b}\left(T-T_{a}\right)+\sigma \varepsilon \left(T^{4}-T_{s}^{4}\right)$$

where *T* represents the temperature, *t* represents time, *k* is the thermal conductivity, $Q_{b}$ is the metabolic heat generation rate, ρ is the density of the tissue, *c_p_* is the specific heat capacity of the tissue, *c_b_* is specific heat capacity of the blood, $\omega_{b}$ is the blood perfusion rate, *T_a_* is the arterial blood temperature, and ∇ represents the gradient operator, σ is the Stefan–Boltzmann constant, *T_s_* is the temperature of the surroundings, and ϵ is the emissivity of the tissue.

Therefore, the recorded temperature changes are related to the temperature gradient, metabolic processes, heat convection through the blood, and radiation, which are influenced by material parameters. The thermal resistance and thermal time constant are values that might also be used in tissue thermal parameterization. They characterize the structure of a given object and its blood supply, as it is related to the effective heat conduction coefficient. Most often, the result is presented as calculated parametric images in the case of ADT, images of the distribution of time constants. When examining the surface of the human body, it should be remembered that the stimulation must have a safe value, i.e., one that will not cause pain and will not damage tissues. Measurement of the surface temperature of a free tissue flap is a popular method of assessing blood flow. Khouri and Shaw [141] performed a retrospective evaluation of 600 cases where surface temperature monitoring was the primary method for evaluation. They defined the coefficient CΔT∘. It represents the change in the temperature difference between the control area and the graft. In their opinion, the difference of 1.8 °C is synonymous with a circulation disorder. Papillion et al. [142], after taking measurements in 47 patients, showed that the CΔT∘ coefficient in the case of the rejected flaps (3.7 °C on average) was about 2 °C higher (*p* > 0.05) than in the case of the accepted grafts. Although skin temperature measurements are believed to be a sensitive and effective tool for examining blood circulation disorders [143], some authors have encountered problems with this method [144]. According to a recent study [145], infrared thermography is a valuable and noninvasive objective tool in free flap monitoring, which can detect flap compromise (increasing value of ΔT) even before it becomes clinically evident. The authors of [146] introduced an experimental method for flap monitoring via infrared thermography by analyzing the temperature differential between the perforator area and the average flap temperature, demonstrating significant potential for enhancing the flap monitoring process.

According to [147], Smartphone-based thermal imaging proved effective, inexpensive, and noninvasive for assessing tissue perfusion, showing promise for predicting flap viability and intraoperative monitoring in head and neck surgery. Smartphone-compatible thermal cameras can be used in addition to clinical examination, as well as other monitoring technologies, providing additional information not only in the selection of perforators in the operating environment, but also in the early diagnosis of the low vitality of parts of the flap, allowing the selective review of areas that would have undergone necrosis [148].

In [149], wireless infrared thermometry device for postoperative flap monitoring was presented and its efficacy in the postoperative monitoring of 40 free flaps was evaluated. The wireless infrared thermometer allowed for earlier detection (30.5 ± 3.1 h) compared and the clinical observation reported (41.7 ± 13.6 h).

#### 3.4.9. Other EM Radiation-Based Methods

Spatial frequency domain imaging is an imaging technique based on tissue absorption and scattering contrast, making it potentially useful for intraoperative assessment of flaps [150]. This approach distinguishes between hemoglobin breakdown products such carboxyhemoglobin and methemoglobin, as well as oxy- and deoxyhemoglobin, by considering both the scatter and spectral characteristics of tissue [151].

Vinogradov and Wilson [152,153,154] were the first to demonstrate the design of synthetic metalloporphyrin-based phosphorescent sensors for oxygen measurements in biological systems. The strong light absorption and phosphorescence emission of the new oxygen sensing phosphors, combined with the ease of converting them into dendrimers for improved compatibility with polymer-based matrices, has allowed the development of oxygen-sensing formulations that can be imaged with portable cameras under ambient room light conditions [155]. In [156], Marks et al. presented the findings of a first-in-human clinical experiment comparing a paintable oxygen-sensing bandage to a standard NIRS-based oximeter for postoperative evaluation of DIEP flaps. The adoption of a transparent oxygen-sensing bandage has two significant advantages. First, it is readily integrated into the present standard of care and does not need substantial training or expertise. Second, the bandage is almost weightless, does not impede the patient’s movement, and does not interfere with visual inspection of the skin tissue beneath.

Recently, the authors of [157] proposed a telemedical technology for condition management of patients in free flap surgery. The system can stream high-quality videos for visual assessment based on an operator-controllable, camera-equipped microcontroller with wi-fi connectivity.

Lee et al. presented the first study to utilize AI based segmentation models for analyzing and evaluation of flaps [158]. The obtained results suggest that AI-based model for flap segmentation and status classification could achieve reliable outcomes.

The authors of the recent study [159] demonstrated the reliability of a machine-learning model in differentiating various types of postoperative flap circulation. Postoperative data from 176 patients who received free flap surgery were prospectively collected, including free flap photographs and clinical evaluation measures. Flap circulation outcome variables included normal, arterial insufficiency, and venous insufficiency. Shapley Additive Explanations were used for prediction interpretations. This novel technique may reduce the burden of free flap monitoring and encourage the broader use of reconstructive microsurgery in regions with a limited number of staff specialists [159]. The deep learning integrated smartphone application can accurately reflect and quantify flap condition. According to the authors of [160] it is a convenient, accurate, and economical device that can improve patient safety and management and assist in monitoring flap physiology.

An experimental research by Zhang et al. [161,162] suggested that Photoacoustic Microscopy could be used as an effective imaging technology platform for the clinical application and scientific research of perforator flaps, including visualizing perforator mapping, predicting the potential necrosis zone of the flap, and observing morphological changes in the choke vessel in a noninvasive and dynamic way.

## 4. Discussion

The article discusses methods for monitoring the viability of post-operative flap monitoring in breast reconstruction. We extensively searched numerous conference and journal publications for a systematic investigation. Irrelevant publications were subsequently excluded, resulting in the retention of 170 papers. Many methods have been used in clinical practice for years. We also review recently implemented methods, and new proposals are still in the pre-clinical trials phase (e.g., ADT). Efficacy, implementation simplicity, and ease of interpretation of measurements have been estimated on the current state of knowledge. In the “simplicity of implementation” criteria, we evaluate the complexity monitoring process, i.e., sensor application or level of expertise needed to operate a particular system. For the “ease of interpretation”, we assess the readability of the measurement and the notability of any vital changes, which are crucial rating factors. The most desirable method should monitor blood flow both in large arteries and at the capillary level in a given tissue volume regardless of the spatial location of the region of interest (ROI). At the moment, such a technique is not achievable. Instead, particular methods are used, and their usefulness is essentially determined by physician experience.

### 4.1. Clinical Monitoring Methods Overview

In Table 2 clinical monitoring techniques were compared. It is worth noting that invasive methods exhibit higher efficacy. On the other hand, their usage is associated with more complicated procedures and, also, the risk of infections is much greater. Furthermore, invasive methods are often associated with higher monitoring costs. Non-invasive methods, on the other hand, are usually characterized by lower efficiency, but also lower application costs. In general, continuous monitoring methods tend to be more expensive; however, more affordable, non-continuous methods require both considerable workload and greater resources. Also, in most cases, non-continuous methods introduce subjectivity of interpretation and larger uncertainty associated with intervals between measurements. The main issue behind currently employed clinical monitoring methods is their poor optimization—systems are often adapted from other fields, and not optimized for monitoring blood flow in the reconstructed breast.

Given that such techniques are often prone to environmental interference a need for the development of a reliable, and accurate system allowing for continuous monitoring of the blood flow in the transplanted tissue is still valid. Methods with a high degree of invasiveness have proven to be more effective, but also considerably more expensive, and not applicable in all cases. Moreover, after the monitoring process, surgical intervention is often required in order to remove the sensor.

Non-invasive methods are often non-continuous or characterized by a low efficacy. Implantable NIRS presented by [106] indicates the potential direction of development of this method–miniaturized, battery supplied, minimally invasive, insensitive to movement device for accurate flap vitality assessment. The Doppler ultrasound device for the continuous monitoring of the absolute velocity of blood flow in deeply embedded arteries based on the Doppler effect has been proposed by Wang et al. [163]. This technique could be adopted for monitoring of blood-flow within tissue flap. Development direction towards combining different modalities, as illustrated by Sentec OxiVenT Sensor, is expected to become more frequent in upcoming years.

Employing Monte-Carlo modeling techniques for photon transport in the tissue could optimize the performance of the NIRS sensor before measurement. Parameters such as light intensity, distance between detector and transmitters, or their arrangement could be altered between patients based on intra-operation observations.

### 4.2. Experimental Monitoring Methods Overview

In Table 3, experimental monitoring techniques were compared. The lack of suitable measuring equipment excludes some promising biochemical monitoring techniques from clinical usage. In its current form, Microdialysis is a precise but labor-intensive and time-consuming method. Perhaps the design of an automated system for continuous measurements could be an interesting solution. Subcutaneous Tissue pH monitoring techniques, despite very short response time, were abandoned by researchers. Potentially design of less invasive (requiring smaller incision) measurement system based on a needle electrode as one presented by Herdman et al. [164] could constitute an interesting development path. The usage of Continuous Glucose Monitors in the assessment of tissue flap vitality seems to be one of the most promising experimental methods. Low cost, quick response time, marginal invasiveness, and suitability for telemedical usage clearly indicates the need for further exploration of the usefulness of this method in clinical applications. A subcutaneous Continuous Lactate Monitor such as the one presented in article by Dror et al. [165] also could play an important role in flap vitality assessment.

Imaging techniques such as Hyperspectral Imaging, Active Dynamic Thermography or image processing present interesting take on flap vitality measurements. One of the most significant advantages of imaging processing techniques is the considerable tolerance of patient movement—limited by the resolution and viewing angle of the camera. The main limitation in their broad application is directly linked to the image acquisition techniques—in the current form, utilized cameras require exposition of larger body parts, which may cause significant discomfort to patients, and is hindered by the use of dressings. Adoption of a transparent oxygen-sensing bandage may partially resolve this issue. The paintable bandage is almost weightless, does not impede the patient’s movement, and does not interfere with visual inspection of the skin tissue beneath. Furthermore, transparent dressing could enable the parallel use of several image processing techniques.

Among the inexpensive and noninvasive methods, magnetic plethysmography (MPG) could be considered. In the literature sensor designs using the Hall sensor and neodymium magnets have been proposed [166,167]. The main differences are in the way blood is magnetized. MPG has not yet been clinically evaluated as a postoperative flap monitoring method, although in the article by Banis et al. [168] similar technique—electromagnetic flowmetry was employed in canine saphenous flap monitoring.

## 5. Conclusions

In comparison to physical examination, most of the presented methods allows for earlier detection of onset complications. In the future, the development of flap monitoring systems with a high degree of reliability and accuracy, allowing for continuous monitoring of the condition of the transplanted tissue, should be considered. Such system should be affordable, efficient, direct, and easy to use on one side, and should enable a spatial scanning of blood perfusion level, in a monitoring mode, on the other side. If possible, reusable probes/sensors should be considered. Currently developed systems should enable consistent measurements to be taken at any time interval and should not be burdensome for the patient. Flap transplant vitality status should be easily monitored by physician on mobile phone, tablet, or watch. If critical values were to be exceeded, the notification should be sent. Furthermore, integration of telemedical solution could potentially shorten the required hospitalization period and cost of care. A non- or slightly invasive techniques should be adopted as a starting point. Potentially new solutions employing optical method to monitor the inflow of oxygenated blood to the area of the transplanted tissue and electrochemical techniques to observe metabolism changes within the area of the transplanted tissue should be considered. Tools employing qualitative analysis to identify areas for future enhancement, such as Flapbot, a promising digital aid for free flap monitoring [169], could be considered by physicians. Image processing techniques and AI based segmentation models for evaluation of flaps are in their early stages. Their intense development in the coming years is expected.

This is a critical review. Limitation of this study is the lack of explicit inclusion and exclusion criteria and a clearly defined process of synthesis. The difficulty in comparing methods is based on the results achieved by various medical centers, employing surgeons with different level of experience, using modifications to well known techniques is worth noting. Postoperative complications and thus the final results often differ from patient to patient. Also, measurement conditions are often omitted, and not well described. This introduces unquantifiable error. As a result, direct comparison of statistics is almost impossible, and makes it difficult to choose the best solution for free flap monitoring. In numerous reports, exclusions from research groups due to complications related to sensor detachment may be observed. Such behaviors tend to produce unrealistic results. There is a need for reliable clinical comparison of currently employed methods. Furthermore, experimental techniques should be also evaluated in comparison to clinically acclaimed methods. 

## Figures and Tables

**Table 1:** The difference between an arterial and a vein occlusion.

	Arterial Supply Obstruction	Venous Outflow Obstruction
Color	Light blue	Purple–blue
Turgor	Lowered	Increased
Capillary refill	Slowed or stopped	Instant
Temperature	Lowered	Increased

**Table 2:** Clinical flap vitality monitoring techniques.

Method	Efficacy	Invasive	Continuous	Direct	Simplicity of Implementation	Ease of Interpretation	Cost	Sensitivity to Movement	Environment Interference Sensitivity
Physical examination.	Low	No	No	No	High	Moderate	n/a	n/a	n/a
Polarography	Moderate	No	Yes	No	High	Moderate	High	High	Low
Handheld Doppler	Moderate	No	No	Yes	High	Moderate	Low	High	Low
Implantable Doppler	Very High	Yes	Yes	Yes	Low	High	Moderate	Moderate	Low
Color Duplex Ultrasography	High	No	No	Yes	Low	Low	Very high	High	Low
Flow Coupler	Very high	Yes	Yes	Yes	Low	Moderate	High	Moderate	Low
Fluorescence Angiography	High	Yes	No	Yes	Moderate	High	Very high	Low	High
PPG	Low	No	Yes	Yes	High	High	Low	Moderate	Moderate
Near-Infrared Spectroscopy	High	No	Yes	Yes	Moderate	High	High	Moderate	Moderate
Laser Doppler Flowmetry	Low	No	Yes	No	High	High	High	Moderate	Moderate
Visible Light Spectroscopy	Moderate	No	Yes	No	High	High	High	Moderate	Moderate

n/a—not applicable.

**Table 3:** Experimental tissue vitality monitoring techniques.

Method	Efficacy	Invasive	Continuous	Direct	Simplicity of Implementation	Ease of Interpretation	Sensitivity to Movement	Environment Interference Sensitivity
Microdialysis	Low	Yes	No	No	Low	High	n/a	n/a
Subcutaneous pH Monitoring	Low	Yes	Yes	No	Moderate	High	Moderate	Moderate
Glucose and lactate Monitoring	Moderate	Yes	No	No	High	High	n/a	n/a
Hyperspectral imaging	Low	No	Yes	No	High	Moderate	Moderate	Moderate
Direct Surface Temperature measurement	Moderate	No	Yes	No	High	High	Low	Low
Active Dynamic Thermography	Moderate	No	Yes	No	Moderate	Moderate	Moderate	Moderate

n/a—not applicable.

## Data Availability

No new data were created or analyzed in this study. Data sharing is not applicable to this article.

## References

[1] Patel U.A., Hernandez D., Shnayder Y., Wax M.K., Hanasono M.M., Hornig J., Ghanem T.A., Old M., Jackson R.S., Ledgerwood L.G. (2017). Free flap reconstruction monitoring techniques and frequency in the era of restricted resident work hours. JAMA-Otolaryngol.-Head Neck Surg..

[2] Chung K. (2019). Grabb and Smith’s Plastic Surgery.

[3] Janis J.E., Kwon R.K., Attinger C.E. (2011). The new reconstructive ladder: Modifications to the traditional model. Plast. Reconstr. Surg..

[4] Rehim S.A., Chung K.C. (2013). Local Flaps of the Hand. Hand Clin..

[5] Mellette J.R., Ho D.Q. (2005). Interpolation flaps. Dermatol. Clin..

[6] Cormack G., Lamberty B. (1984). A classification of fascio-cutaneous flaps according to their patterns of vascularisation. Br. J. Plast. Surg..

[7] Gagnon A., Blondeell P. (2006). Deep and superficial inferior epigastric artery perforator flaps. Cirug. Plástica-Ibero-Latinoam..

[8] Knox A.D.C., Ho A.L., Leung L., Tashakkor A.Y., Lennox P.A., Van Laeken N., Macadam S. (2016). Comparison of Outcomes following Autologous Breast Reconstruction Using the DIEP and Pedicled TRAM Flaps. Plast. Reconstr. Surg..

[9] Taylor G.I., Palmer J.H. (1987). The vascular territories (angiosomes) of the body: Experimental study and clinical applications. Br. J. Plast. Surg..

[10] Saint-Cyr M., Wong C., Schaverien M., Mojallal A., Rohrich R.J. (2009). The perforasome theory: Vascular anatomy and clinical implications. Plast. Reconstr. Surg..

[11] Taylor G.I., Chubb D.P., Ashton M.W. (2013). True and “choke” anastomoses between perforator angiosomes: Part I. Anatomical location. Plast. Reconstr. Surg..

[12] Saint-Cyr M., Schaverien M., Wong C., Nagarkar P., Arbique G., Brown S., Rohrich R.J. (2009). The extended anterolateral thigh flap: Anatomical basis and clinical experience. Plast. Reconstr. Surg..

[13] Kubo T., Yano K., Hosokawa K. (2002). Management of flaps with compromised venous outflow in head and neck microsurgical reconstruction. Microsurgery.

[14] Mao C., Yu G.Y., Peng X., Guo C.B., Huang M.X. (2005). Postoperative vessel thrombosis and its management after free flap transfers in head and neck region. Zhonghua Er Bi Yan Hou Tou Jing Wai Ke Za Zhi.

[15] Paydarfar J.A., Birkmeyer N.J. (2006). Complications in Head and Neck Surgery. Arch. Otolaryngol. Neck Surg..

[16] Fearon J.A., Cuadros C.L., May J.W. (1990). Flap failure after microvascular free-tissue transfer: The fate of a second attempt. Plast. Reconstr. Surg..

[17] Devine J.C., Potter L.A., Magennis P., Brown J.S., Vaughan E.D. (2001). Flap Monitoring after Head and Neck Reconstruction: Evaluating an Observation Protocol. J. Wound Care.

[18] Brown J.S., Devine J.C., Magennis P., Sillifant P., Rogers S.N., Vaughan E.D. (2003). Factors that Influence the Outcome of Salvage in Free Tissue Transfer. Br. J. Oral Maxillofac. Surg..

[19] Bianchi B., Copelli C., Ferrari S., Ferri A., Sesenna E. (2009). Free flaps: Outcomes and complications in head and neck reconstructions. J.-Cranio-Maxillofac. Surg..

[20] Yu P., Chang D.W., Miller M.J., Reece G., Robb G.L. (2009). Analysis of 49 cases of flap compromise in 1310 free flaps for head and neck reconstruction. Head Neck.

[21] Yang J.C.S., Kuo Y.R., Hsieh C.H., Jeng S.F. (2007). The use of radial vessel stump in free radial forearm flap as flap monitor in head and neck reconstruction. Ann. Plast. Surg..

[22] Pohlenz P., Klatt J., Schön G., Blessmann M., Li L., Schmelzle R. (2012). Microvascular free flaps in head and neck surgery: Complications and outcome of 1000 flaps. Int. J. Oral Maxillofac. Surg..

[23] Weinzweig N., Lukash F., Weinzweig J. (1999). Topical and systemic calcium channel blockers in the prevention and treatment of microvascular spasm in a rat epigastric island skin flap model. Ann. Plast. Surg..

[24] Jernbeck J., Samuelson U. (1993). Effects of Lidocaine and Calcitonin Gene-Related Peptide (CGRP) on Isolated Human Radial Arteries. J. Reconstr. Microsurg..

[25] Komorowska-Timek E., Chen S.G., Zhang F., Dogan T., Lineaweaver W.C., Buncke H.J. (1999). Prolonged perivascular use of verapamil or lidocaine decreases skin flap necrosis. Ann. Plast. Surg..

[26] Kuo L., Davis M., Chilian W. (1992). Endothelial Modulation of Arteriolar Tone. Physiology.

[27] Wettstein R., Wessendorf R., Sckell A., Leunig M., Banic A., Erni D. (2000). The Effect of Pedicle Artery Vasospasm on Microhemodynamics in Anatomically Perfused and Extended Skin Flap Tissue. Ann. Plast. Surg..

[28] Bui D.T., Cordeiro P.G., Hu Q.Y., Disa J.J., Pusic A., Mehrara B.J. (2007). Free flap reexploration: Indications, treatment, and outcomes in 1193 free flaps. Plast. Reconstr. Surg..

[29] Glass G.E., Nanchahal J. (2012). Why haematomas cause flap failure: An evidence-based paradigm. J. Plast. Reconstr. Aesthetic Surg..

[30] Ahmad F.I., Gerecci D., Gonzalez J.D., Peck J.J., Wax M.K. (2015). The role of postoperative hematoma on free flap compromise. Laryngoscope.

[31] Kaplan E.D., Rozen W.M., Shayan R., Bernard S., Macgill K. (2008). Preventing postoperative haematomas in microvascular reconstruction of the head and neck: Lessons learnt from 126 consecutive cases. ANZ J. Surg..

[32] Kroll S.S., Miller M.J., Reece G.P., Baldwin B.J., Robb G.L., Bengtson B.P. (1995). Anticoagulants and hematomas in free flap surgery. Plast. Reconstr. Surg..

[33] Zoccali G., Molina A., Farhadi J. (2017). Is long-term post-operative monitoring of microsurgical flaps still necessary?. J. Plast. Reconstr. Aesthetic Surg..

[34] Novakovic D., Patel R.S., Goldstein D.P., Gullane P.J. (2009). Salvage of failed free flaps used in head and neck reconstruction. Head Neck Oncol..

[35] Shen A.Y., Lonie S., Lim K., Farthing H., Hunter-Smith D.J., Rozen W.M. (2021). Free flap monitoring, salvage, and failure timing: A systematic review. J. Reconstr. Microsurg..

[36] Smit J.M., Zeebregts C.J., Acosta R. (2008). Timing of Presentation of the First Signs of Vascular Compromise Dictates the Salvage Outcome of Free Flap Transfers. Plast. Reconstr. Surg..

[37] Yang Q., Ren Z.H., Chickooree D., Wu H.J., Tan H.Y., Wang K., He Z.J., Gong C.J., Ram V., Zhang S. (2014). The Effect of Early Detection of Anterolateral Thigh Free Flap Crisis on the Salvage Success Rate, Based on 10 Years of Experience and 1072 Flaps. Int. J. Oral Maxillofac. Surg..

[38] Ho M.W., Brown J.S., Magennis P., Bekiroglu F., Rogers S.N., Shaw R.J., Vaughan E.D. (2012). Salvage Outcomes of Free Tissue Transfer in Liverpool: Trends Over 18 Years (1992–2009). Br. J. Oral Maxillofac. Surg..

[39] Kroll S.S., Schusterman M.A., Reece G.P., Miller M.J., Evans G.R., Robb G.L., Baldwin B.J. (1996). Timing of pedicle thrombosis and flap loss after free-tissue transfer. Plast. Reconstr. Surg..

[40] Hirigoyen M.B., Urken M.L., Weinberg H. (1995). Free Flap Monitoring: A Review of Current Practice. Microsurgery.

[41] Lovětínská V., Sukop A., Klein L., Brandejsova A. (2020). Free-flap monitoring: Review and clinical approach. Acta Chir. Plast..

[42] Vijan S.S., Tran N.V. (2007). Microvascular breast reconstruction pedicle thrombosis: How long can we wait?. Microsurg. Off. J. Int. Microsurg. Soc. Eur. Fed. Soc. Microsurg..

[43] Du W., Wu P.F., Qing L.M., Wang C.Y., Liang J.Y., Yu F., Tang J.-Y. (2015). Systemic and Flap Inflammatory Response Associates with Thrombosis in Flap Venous Crisis. Inflammation.

[44] Anagnos V.J., Brody R.M., Carey R.M., De Ravin E., Tasche K.K., Newman J.G., Shanti R.M., Chalian A.A., Rassekh C.H., Weinstein G.S. (2023). Post-operative monitoring for head and neck microvascular reconstruction in the era of resident duty hour restrictions: A retrospective cohort study comparing 2 monitoring protocols. Ann. Otol. Rhinol. Laryngol..

[45] Heyrovskỳ J., Shikata M. (1925). Researches with the dropping mercury cathode: Part II. The Polarograph. Recl. Trav. Chim.-Pays-Bas.

[46] Baumberger J., Goodfriend R. (1951). Determination of Arterial Oxygen Tension in Man by Equilibration Through Intact Skin.

[47] Clark L.C., Wolf R., Granger D., Taylor Z. (1953). Continuous recording of blood oxygen tensions by polarography. J. Appl. Physiol..

[48] Huch R., Lübbers D., Huch A. (1972). Quantitative continuous measurement of partial oxygen pressure on the skin of adults and new-born babies. Pflügers Archiv..

[49] Chang N., Goodson W.H., Gottrupp F., Hunt T.K. (1983). Direct Measurement of Wound and Tissue Oxygen Tension in Postoperative Patients. Ann. Surg..

[50] Kamolz L.P., Giovanoli P., Haslik W., Koller R., Frey M. (2002). Continuous free-flap monitoring with tissue-oxygen measurements: Three-year experience. J. Reconstr. Microsurg..

[51] Wechselberger G., Rumer A., Schoeller T., Schwabegger A., Ninkovic M., Anderl H. (1997). Free-flap monitoring with tissue-oxygen measurement. J. Reconstr. Microsurg..

[52] Jonas R., Schaal T., Krimmel M., Gülicher D., Reinert S., Hoffmann J. (2013). Monitoring in microvascular tissue transfer by measurement of oxygen partial pressure: Four years experience with 125 microsurgical transplants. J.-Cranio-Maxillofac. Surg..

[53] Raittinen L., Pukander J., Laranne J. (2012). Early recognition of ischaemia with continuous real-time tissue oxygen monitoring in head and neck microvascular flaps. Eur. J. Plast. Surg..

[54] Arnež Z.M., Ramella V., Papa G., Novati F.C., Manca E., Leuzzi S., Stocco C. (2019). Is the LICOX^®^ PtO_2_ system reliable for monitoring of free flaps? Comparison between two cohorts of patients. Microsurgery.

[55] Trignano E., Fallico N., Fiorot L., Bolletta A., Maffei A., Ciudad P., Maruccia M., Chen H.-C. (2017). Flap Monitoring with Continuous Oxygen Partial Tension Measurement in Breast Reconstructive Surgery: A Preliminary Report. Microsurgery.

[56] Severinghaus J.W. (1960). Methods of measurement of blood and gas carbon dioxide during anesthesia. J. Am. Soc. Anesthesiol..

[57] Johns R., Lindsay W., Shepard R. (1969). A system for monitoring pulmonary ventilation. Biomed. Sci. Instrum..

[58] Van Weteringen W., Goos T.G., Van Essen T., Ellenberger C., Hayoz J., De Jonge R.C., Reiss I.K., Schumacher P.M. (2020). Novel transcutaneous sensor combining optical tcPO_2_ and electrochemical tcPCO_2_ monitoring with reflectance pulse oximetry. Med. Biol. Eng. Comput..

[59] Delgado J., DeFeudis F., Roth R., Ryugo D., Mitruka B. (1972). Dialytrode for Long Term Intracerebral Perfusion in Awake Monkeys. Arch. Int. Pharmacodyn. Thér..

[60] Setälä L.P., Korvenoja E.M., Härmä M.A., Alhava E.M., Uusaro A.V., Tenhunen J.J. (2004). Glucose, Lactate, and Pyruvate Response in an Experimental Model of Microvascular Flap Ischemia and Reperfusion: A Microdialysis Study. Microsurgery.

[61] Setala L., Gudaviciene D. (2013). Glucose and lactate metabolism in well-perfused and compromised microvascular flaps. J. Reconstr. Microsurg..

[62] Goodman J.C., Valadka A.B., Gopinath S.P., Uzura M., Robertson C.S. (1998). Clinical Microdialysis in the Neurosurgical Intensive Care Unit. Crit. Care Med..

[63] Setälä L.P., Papp A., Romppanen E.L., Mustonen P., Berg L., Härmä M. (2006). Microdialysis Detects Postoperative Perfusion Failure in Microvascular Flaps. J. Reconstr. Microsurg..

[64] Hwang S.J. (2023). Diagnostic Accuracy of Microdialysis in Postoperative Flap Monitoring. J. Craniofacial Surg..

[65] Sorotos M., Firmani G., Tornambene R., Marrella D., Paolini G., Santanelli di Pompeo F. (2024). DIEP flap perfusion assessment using microdialysis versus Doppler ultrasonography: A comparative study. Microsurgery.

[66] Jyranki J., Suominen S., Vuola J., Back L. (2006). Microdialysis in Clinical Practice. Ann. Plast. Surg..

[67] Mourouzis C., Anand R., Bowden J.R., Brennan P.A. (2007). Microdialysis: Use in the assessment of a buried bone-only fibular free flap. Plast. Reconstr. Surg..

[68] Dakpé S., Colin E., Bettoni J., Davrou J., Diouf M., Devauchelle B., Testelin S. (2020). Intraosseous microdialysis for bone free flap monitoring in head and neck reconstructive surgery: A prospective pilot study. Microsurgery.

[69] Dickson M.G., Sharpe D.T. (1985). Continuous Subcutaneous Tissue pH Measurement as a Monitor of Blood Flow in Skin Flaps: An Experimental Study. Br. J. Plast. Surg..

[70] Hedén P., Sollevi A. (1989). Circulatory and metabolic events in pig island skin flaps after arterial or venous occlusion. Plast. Reconstr. Surg..

[71] Singh K., Shah S., Mittal R.K., Garg R. (2023). Role of Lactate Measurement in Flap Monitoring: An Useful Adjunct. Indian J. Plast. Surg..

[72] Sakakibara S., Hashikawa K., Omori M., Terashi H., Tahara S. (2010). A simplest method of flap monitoring. J. Reconstr. Microsurg..

[73] Tachi K., Nakatsukasa S., Nakayama Y. (2018). Monitoring free flap venous congestion using continuous tissue glucose monitoring: A case report. JPRAS Open.

[74] Kishi K., Ishida K., Makino Y., Miyawaki T. (2019). A simple way to measure glucose and lactate values during free flap head and neck reconstruction surgery. J. Oral Maxillofac. Surg..

[75] Kiuchi T., Ishii N., Uno T., Uoya Y., Sakai S., Matsuzaki K., Kishi K. (2024). Flap Monitoring Using Interstitial Fluid Glucose Measurements. Plast. Surg..

[76] Colman M.P., Schauvinhold C., Chavanne J., Errea G., Bou M., Ernst G. (2023). DIY Flap Monitoring: The Glucose Index. Plast. Reconstr.-Surg.-Glob. Open.

[77] Onoda S., Satake T., Katsuragi R., Kobayashi K., Tsukura K., Tachibana G. (2023). A Novel Doppler Flowmetry Shaft for Postoperative Monitoring after Head and Neck Reconstructive Surgery. Plast. Reconstr.-Surg.-Glob. Open.

[78] Hallock G.G. (2011). Acoustic Doppler Sonography, Color Duplex Ultrasound, and Laser Doppler Flowmetry as Tools for Successful Autologous Breast Reconstruction. Clin. Plast. Surg..

[79] Fujiwara R.J.T., Dibble J.M., Larson S.V., Pierce M.L., Mehra S. (2018). Outcomes and Reliability of the Flow Coupler in Postoperative Monitoring of Head and Neck Free Flaps. Laryngoscope.

[80] Hosein R.C., Cornejo A., Wang H.T. (2016). Postoperative Monitoring of Free Flap Reconstruction: A Comparison of External Doppler Ultrasonography and the Implantable Doppler Probe. Plast. Surg..

[81] Poder T.G., Fortier P.H. (2013). Implantable Doppler in Monitoring Free Flaps: A Cost-Effectiveness Analysis Based on a Systematic Review of the Literature. Eur. Ann. Otorhinolaryngol. Head Neck Dis..

[82] Dunklebarger M.F., McCrary H., King B., Carpenter P., Buchmann L., Hunt J., Cannon R. (2022). Success of implantable doppler probes for monitoring buried free flaps. Otolaryngol.-Head Neck Surg..

[83] Tabrizi R., Okhovatpour M., Hassani M., Rashad A. (2021). Comparison of Single Photon Emission Computed Tomography (SPECT) and implantable Doppler in the monitoring of a vascularised fibular free flap for reconstruction of the mandible. Br. J. Oral Maxillofac. Surg..

[84] Cervenka B., Bewley A.F. (2015). Free Flap Monitoring. Curr. Opin. Otolaryngol. Head Neck Surg..

[85] Hayler R., Low T.H., Fung K., Nichols A.C., MacNeil S.D., Yoo J. (2021). Implantable Doppler ultrasound monitoring in head and neck free flaps: Balancing the pros and cons. Laryngoscope.

[86] Obana A., Miki T., Hayashi K., Takeda M., Kawamura A., Mutoh T., Harino S., Fukushima I., Komatsu H., Takaku Y. (1994). Survey of Complications of Indocyanine Green Angiography in Japan. Am. J. Ophthalmol..

[87] Holm C., Tegeler J., Mayr M., Becker A., Pfeiffer U., Mühlbauer W. (2002). Monitoring free flaps using laser-induced fluorescence of indocyanine green: A preliminary experience. Microsurg. Off. J. Int. Microsurg. Soc. Eur. Fed. Soc. Microsurg..

[88] Krishnan K.G., Schackert G., Steinmeier R. (2005). The role of near-infrared angiography in the assessment of post-operative venous congestion in random pattern, pedicled island and free flaps. Br. J. Plast. Surg..

[89] Hitier M., Cracowski J.L., Hamou C., Righini C., Bettega G. (2016). Indocyanine green fluorescence angiography for free flap monitoring: A pilot study. J. -Cranio-Maxillofac. Surg..

[90] Adelsberger R., Fakin R., Mirtschink S., Forster N., Giovanoli P., Lindenblatt N. (2019). Bedside monitoring of free flaps using ICG-fluorescence angiography significantly improves detection of postoperative perfusion impairment#. J. Plast. Surg. Hand Surg..

[91] Yang H., Kim J., Nam W., Kim H.J., Cha I.h., Kim D. (2021). Handheld near-infrared fluorescence imaging device using modified action cameras for peri-operative guidance of microvascular flap surgery. J. Clin. Med..

[92] Schoepper S., Smeets R., Gosau M., Hanken H. (2022). Intraoperative ICG-based fluorescence-angiography in head and neck reconstruction: Predictive value for impaired perfusion of free flaps. J.-Cranio-Maxillofac. Surg..

[93] Hoven P.V.D., Verduijn P.S., Capelle L.V., Tange F.P., Michi M., Corion L.U.M., Mulder B.S., Mureau M.A.M., Vahrmeijer A.L., Van Der Vorst J.R. (2022). Quantification of Near-infrared Fluorescence Imaging with Indocyanine Green in Free Flap Breast Reconstruction. J. Plast. Reconstr. Aesthetic Surg..

[94] Taghizadeh F., Troob S.H., Wax M.K. (2023). The role of fluorescent angiography in free flap reconstruction of the head and neck. Laryngoscope.

[95] Brinkman R., Zylstra W. (1949). Determination and continuous registration of the percentage oxygen saturation in clinical conditions. Arch. Chir. Neerl..

[96] Futran N.D., Stack B.C., Hollenbeak C., Scharf J.E. (2000). Green light photoplethysmography monitoring of free flaps. Arch.-Otolaryngol.-Head Neck Surg..

[97] Kyriacou P., Zaman T., Pal S. (2020). Photoplethysmogrphy in postoperative monitoring of deep inferior epigastric perforator (DIEP) free flaps. Physiol. Meas..

[98] Delpy D.T., Ferrari M., Piantadosi C.A., Tamura M. (2007). Pioneers in biomedical optics: Special section honoring Professor Frans F. Jöbsis of Duke University. J. Biomed. Opt..

[99] Jöbsis F.F. (1977). Noninvasive, infrared monitoring of cerebral and myocardial oxygen sufficiency and circulatory parameters. Science.

[100] Repež A., Oroszy D., Arnež Z.M. (2008). Continuous Postoperative Monitoring of Cutaneous Free Flaps Using Near Infrared Spectroscopy. J. Plast. Reconstr. Aesthetic Surg..

[101] Ma Y., Li S., Shan X., Zhang L., Cai Z. (2024). Continuous Monitoring of Buried Free Bone Flap Microcirculation Using a Near-Infrared Spectroscopy System. Plast. Reconstr. Surg..

[102] Steenhaut K., Bove T., De Hert S., Moerman A. (2017). Comparison of Three NIRS Devices for the Measurement of Microvascular Reactivity.

[103] Lin C.M., Huang C.C., Hsu H., Chiu C.H., Chien S.H. (2010). Advancements in free flap monitoring in the last decade: A critical review. Plast. Reconstr. Surg..

[104] Yuen J.C. (2019). Comparison between near-infrared spectroscopy and laser Doppler flowmetry in free flap adjunct monitoring. Plast. Reconstr.-Surg.-Glob. Open.

[105] Schoenbrunner A., Hackenberger P.N., DeSanto M., Chetta M. (2021). Cost-effectiveness of vioptix versus clinical examination for flap monitoring of autologous free tissue breast reconstruction. Plast. Reconstr. Surg..

[106] Guo H., Bai W., Ouyang W., Liu Y., Wu C., Xu Y., Weng Y., Zang H., Liu Y., Jacobson L. (2022). Wireless implantable optical probe for continuous monitoring of oxygen saturation in flaps and organ grafts. Nat. Commun..

[107] Czako L., Simko K., Sovis M., Vidova I., Sufliarsky B., Odnoga P., Galis B. (2023). Near infrared spectroscopy in monitoring of head and neck microvascular free flaps. Bratisl. Med. J./Bratisl. Lekárske Listy.

[108] Malagón P., Taghizadeh R., Torrano L., González J. (2023). A new protocol for improving immediate monitoring of skin-island free flap with near-infrared spectroscopy and ultrasound. J. Plast. Reconstr. Aesthetic Surg..

[109] Harper J., Slade E., Cornette A., Kejner A.E. (2024). Second sensor to improve near-infrared spectroscopy flap monitor utility: A prospective study. Microsurgery.

[110] Limjeerajarus C. (2014). Laser Doppler Flowmetry: Basic Principle, Current Clinical and Research Applications in Dentistry. CU Dent. J..

[111] Tschumi C., Seyed Jafari S.M., Rothenberger J., Van de Ville D., Keel M., Krause F., Shafighi M. (2015). Post-operative monitoring of free muscle transfers by Laser Doppler Imaging: A prospective study. Microsurgery.

[112] Fredriksson I., Fors C., Johansson J. (2007). Laser Doppler Flowmetry—A Theoretical Framework.

[113] Hölzle F., Loeffelbein D.J., Nolte D., Wolff K.D. (2006). Free Flap Monitoring Using Simultaneous Non-invasive Laser Doppler Flowmetry and Tissue Spectrophotometry. J.-Cranio-Maxillofac. Surg..

[114] Heller L., Levin L.S., Klitzman B. (2001). Laser Doppler Flowmeter Monitoring of Free Tissue Transfers: Blood Flow in Normal and Complicated Cases. Plast. Reconstr. Surg..

[115] Ooms M., Winnand P., Heitzer M., Peters F., Bock A., Katz M.S., Hölzle F., Modabber A. (2023). Attached compared with unattached surface probes for monitoring flap perfusion in microvascular head and neck reconstruction: A feasibility study. Sci. Rep..

[116] Benaron D.A., Parachikov I.H., Cheong W.F., Friedland S., Rubinsky B.E., Otten D.M., Liu F.W., Levinson C.J., Murphy A.L., Price J.W. (2005). Design of a visible-light spectroscopy clinical tissue oximeter. J. Biomed. Opt..

[117] Karliczek A., Benaron D., Baas P., Zeebregts C., Wiggers T., Van Dam G. (2010). Intraoperative assessment of microperfusion with visible light spectroscopy for prediction of anastomotic leakage in colorectal anastomoses. Color. Dis..

[118] Ho J.K., Liakopoulos O.J., Crowley R., Yezbick A.B., Sanchez E., Shivkumar K., Mahajan A. (2009). In vivo detection of myocardial ischemia in pigs using visible light spectroscopy. Anesth. Analg..

[119] Cornejo A., Rodriguez T., Steigelman M., Stephenson S., Sahar D., Cohn S.M., Michalek J.E., Wang H.T. (2011). The use of visible light spectroscopy to measure tissue oxygenation in free flap reconstruction. J. Reconstr. Microsurg..

[120] Fox P.M., Zeidler K., Carey J., Lee G.K. (2013). White light spectroscopy for free flap monitoring. Microsurgery.

[121] Moreno-Oyervides A., Díaz-Ojeda L., Bonilla-Manrique O.E., Bonastre-Juliá J., Largo-Aramburu C., Acedo P., Martín-Mateos P. (2023). Design and testing of an optical instrument for skin flap monitoring. Sci. Rep..

[122] Lu G., Fei B. (2014). Medical hyperspectral imaging: A review. J. Biomed. Opt..

[123] Marotz J., Siafliakis A., Holmer A., Kulcke A., Siemers F. (2015). First results of a new hyperspectral camera system for chemical based wound analysis. Wound Med..

[124] Holmer A., Tetschke F., Marotz J., Malberg H., Markgraf W., Thiele C., Kulcke A. (2016). Oxygenation and perfusion monitoring with a hyperspectral camera system for chemical based tissue analysis of skin and organs. Physiol. Meas..

[125] Kulcke A., Holmer A., Wahl P., Siemers F., Wild T., Daeschlein G. (2018). A compact hyperspectral camera for measurement of perfusion parameters in medicine. Biomed. Eng. Tech..

[126] Thiem D.G., Römer P., Blatt S., Al-Nawas B., Kämmerer P.W. (2021). New approach to the old challenge of free flap monitoring—Hyperspectral imaging outperforms clinical assessment by earlier detection of perfusion failure. J. Pers. Med..

[127] Kohler L.H., Köhler H., Kohler S., Langer S., Nuwayhid R., Gockel I., Spindler N., Osterhoff G. (2021). Hyperspectral Imaging (HSI) as a new diagnostic tool in free flap monitoring for soft tissue reconstruction: A proof of concept study. BMC Surg..

[128] Campbell J.M., Habibalahi A., Handley S., Agha A., Mahbub S.B., Anwer A.G., Goldys E.M. (2023). Emerging clinical applications in oncology for non-invasive multi-and hyperspectral imaging of cell and tissue autofluorescence. J. Biophotonics.

[129] Thiem D.G., Römer P., Gielisch M., Al-Nawas B., Schlüter M., Plaß B., Kämmerer P.W. (2021). Hyperspectral imaging and artificial intelligence to detect oral malignancy–part 1-automated tissue classification of oral muscle, fat and mucosa using a light-weight 6-layer deep neural network. Head Face Med..

[130] Merdasa A., Berggren J., Tenland K., Stridh M., Hernandez-Palacios J., Gustafsson N., Sheikh R., Malmsjö M. (2023). Oxygen saturation mapping during reconstructive surgery of human forehead flaps with hyperspectral imaging and spectral unmixing. Microvasc. Res..

[131] Chen K.T., Mardini S., Chuang D.C., Lin C., Cheng M.H., Lin Y.T., Huang W.C., Tsao C.K., Wei F.C. (2007). Timing of Presentation of the First Signs of Vascular Compromise Dictates the Salvage Outcome of Free Flap Transfers. Plast. Reconstr. Surg..

[132] Zhao Y., Bergmann J.H.M. (2023). Non-Contact Infrared Thermometers and Thermal Scanners for Human Body Temperature Monitoring: A Systematic Review. Sensors.

[133] Avraham M., Nemirovsky J., Blank T., Golan G., Nemirovsky Y. (2022). Toward an Accurate IR Remote Sensing of Body Temperature Radiometer Based on a Novel IR Sensing System Dubbed Digital TMOS. Micromachines.

[134] Lawson R. (1956). Implications of Surface Temperatures in the Diagnosis of Breast Cancer. Can. Med. Assoc. J..

[135] Yamamoto T., Todokoro T., Koshima I. (2012). Handheld Thermography for Flap Monitoring. J. Plast. Reconstr. Aesthetic Surg..

[136] Moderhak M., Kaczmarek M. (2019). Thermal Sequences Database of the Skin Flaps in Breast Reconstruction and Burns. Meas. Autom. Monit..

[137] Kolacz S., Moderhak M., Jankau J. (2017). New Perspective on the In Vivo Use of Cold Stress Dynamic Thermography in Integumental Reconstruction with the Use of Skin-Muscle Flaps. J. Surg. Res..

[138] Kaczmarek M., Nowakowski A. (2016). Active IR-Thermal Imaging in Medicine. J. Nondestruct. Eval..

[139] Kaczmarek M., Nowakowski A. (2017). Active dynamic thermography in medical diagnostics. Application of Infrared to Biomedical Sciences.

[140] Pennes H.H. (1948). Analysis of tissue and arterial blood temperatures in the resting human forearm. J. Appl. Physiol..

[141] Khouri R.K., Shaw W.W. (1992). Monitoring of Free Flaps with Surface-Temperature Recordings. Plast. Reconstr. Surg..

[142] Papillion P., Wong L., Waldrop J., Sargent L., Brzezienski M. (2009). Infrared Surface Temperature Monitoring in the Postoperative Management of Free Tissue Transfers. Plast. Surg..

[143] Levinsohn D.G., Gordon L., Sessler D.I. (1991). Comparison of Four Objective Methods of Monitoring Digital Venous Congestion. J. Hand Surg..

[144] Busic V., Das-Gupta R. (2004). Temperature Monitoring in Free Flap Surgery. Br. J. Plast. Surg..

[145] Singla P., Dixit P.K., Kala P.C., Katrolia D., Karmakar S., Humnekar A., Singh A.P. (2024). Free Flap Monitoring Using Infrared Thermography: An Objective Adjunct to Clinical Monitoring. Indian J. Plast. Surg..

[146] Kim H., Kwak S.H., Byeon J.Y., Lee D.W., Kim J.H., Lim S., Choi H.J. (2024). An Experimental and Clinical Study of Flap Monitoring with an Analysis of the Clinical Course of the Flap Using an Infrared Thermal Camera. Bioengineering.

[147] Fiedler L.S., Lippert B.M., Adrian L., Meyer T. (2024). Perfusion in Pedicled Skin Flaps: Initial Insights from Smartphone-Based Thermal Imaging Protocol. J. Pers. Med..

[148] Vaccari S., Lorenzano V., Lisa A., Di Giuli R., Klinger M., Klinger F., Vinci V. (2023). Smartphone Dynamic Infrared Thermography for Harvesting AICAP Flap in a Large Breast-conservative Surgery. Plast. Reconstr.-Surg.-Glob. Open.

[149] Xie R., Zhang Y., Liu Q., Huang X., Liu M. (2023). A wireless infrared thermometry device for postoperative flap monitoring: Proof of concept in patients. Surg. Innov..

[150] Nguyen J.T., Lin S.J., Tobias A.M., Gioux S., Mazhar A., Cuccia D.J., Ashitate Y., Stockdale A., Oketokoun R., Durr N.J. (2013). A novel pilot study using spatial frequency domain imaging to assess oxygenation of perforator flaps during reconstructive breast surgery. Ann. Plast. Surg..

[151] Saager R.B., Rowland R.A., Baldado M.L., Kennedy G.T., Bernal N.P., Ponticorvo A., Christy R.J., Durkin A.J. (2019). Impact of hemoglobin breakdown products in the spectral analysis of burn wounds using spatial frequency domain spectroscopy. J. Biomed. Opt..

[152] Vinogradov S.A., Wilson D.F. (1995). Metallotetrabenzoporphyrins. New phosphorescent probes for oxygen measurements. J. Chem. Soc. Perkin Trans. 2.

[153] Vinogradov S.A., Wilson D.F. (1998). Phosphorescent Dendritic Macromolecular Compounds for Imaging Tissue Oxygen. U.S. Patent.

[154] Wilson D. (1993). Measuring oxygen using oxygen dependent quenching of phosphorescence: A status report. Optical Imaging of Brain Function and Metabolism. Advances in Experimental Medicine and Biology.

[155] Roussakis E., Li Z., Nowell N.H., Nichols A.J., Evans C.L. (2015). Bright,“clickable” porphyrins for the visualization of oxygenation under ambient light. Angew. Chem. Int. Ed..

[156] Marks H., Bucknor A., Roussakis E., Nowell N., Kamali P., Cascales J.P., Kazei D., Lin S.J., Evans C.L. (2020). A paintable phosphorescent bandage for postoperative tissue oxygen assessment in DIEP flap reconstruction. Sci. Adv..

[157] Yoon K., Cho S.M., Kim K.G., Cheon Y.W. (2023). Remote Monitoring System for Condition Management of Patients in Free Flap Surgery. Surg. Innov..

[158] Lee S.M., Chung M.J., Kim Z., Lee K.T., Kim J.S. Automatic Segmentation and Evaluation Techniques for Free Flap in Reconstruction Surgery Using Deep Learning. Proceedings of the 2023 IEEE Conference on Artificial Intelligence (CAI).

[159] Huang R.W., Tsai T.Y., Hsieh Y.H., Hsu C.C., Chen S.H., Lee C.H., Lin Y.T., Kao H.K., Lin C.H. (2023). Reliability of postoperative free flap monitoring with a novel prediction model based on supervised machine learning. Plast. Reconstr. Surg..

[160] Hsu S.Y., Chen L.W., Huang R.W., Tsai T.Y., Hung S.Y., Cheong D.C.F., Lu J.C.Y., Chang T.N.J., Huang J.J., Tsao C.K. (2023). Quantization of extraoral free flap monitoring for venous congestion with deep learning integrated iOS applications on smartphones: A diagnostic study. Int. J. Surg..

[161] Zhang D., Chen H., Hu X., Yu A. (2021). Photoacoustic microscopy: A novel approach for studying perforator skin flap in a mouse model. Quant. Imaging Med. Surg..

[162] Zhang D., Yuan Y., Zhang H., Yi X., Xiao W., Yu A. (2021). Photoacoustic microscopy provides early prediction of tissue necrosis in skin avulsion injuries. Clin. Cosmet. Investig. Dermatol..

[163] Wang F., Jin P., Feng Y., Fu J., Wang P., Liu X., Zhang Y., Ma Y., Yang Y., Yang A. (2021). Flexible Doppler ultrasound device for the monitoring of blood flow velocity. Sci. Adv..

[164] Herdman K.M., Breslin C.B., Finnerty N.J. (2019). Physiological monitoring of tissue pH: In vitro characterisation and in vivo validation of a quinone-modified carbon paste electrode. Electrochim. Acta.

[165] Dror N., Weidling J., White S., Ortenzio F., Shreim S., Keating M.T., Pham H., Radom-Aizik S., Botvinick E. (2021). Clinical evaluation of a novel subcutaneous lactate monitor. J. Clin. Monit. Comput..

[166] Nabeel P.M., Joseph J., Sivaprakasam M. (2017). A Magnetic Plethysmograph Probe for Local Pulse Wave Velocity Measurement. IEEE Trans. Biomed. Circuits Syst..

[167] Chandrasekhar A., Joseph J., Sivaprakasam M. Local Pulse Wave Velocity Estimation using Magnetic Plethysmograph. Proceedings of the 2013 35th Annual International Conference of the IEEE Engineering in Medicine and Biology Society (EMBC).

[168] Banis J.C., Schwartz K.S., Acland R.D. (1980). Electromagnetic flowmetry—An experimental method for continuous blood flow measurement using a new island flap model. Plast. Reconstr. Surg..

[169] Ejaz H., Ali S.R., Berner J.E., Dobbs T., Whitaker I.S. (2024). The ‘Flapbot’—A global perspective on the validity and usability of a flap monitoring chatbot. J. Reconstr. Microsurg..

